# Asymmetric Ugi 3CR on isatin-derived ketimine: synthesis of chiral 3,3-disubstituted 3-aminooxindole derivatives

**DOI:** 10.3762/bjoc.10.141

**Published:** 2014-06-18

**Authors:** Giordano Lesma, Fiorella Meneghetti, Alessandro Sacchetti, Mattia Stucchi, Alessandra Silvani

**Affiliations:** 1 Dipartimento di Chimica, Università degli Studi di Milano, via C. Golgi, 19, 20133 Milano (Italy); 2 Dipartimento di Scienze Farmaceutiche, Università degli Studi di Milano, via L. Mangiagalli 25, 20133 Milano, Italy; 3 Dipartimento di Chimica, Materiali e Ingegneria Chimica ‘Giulio Natta’, Politecnico di Milano, p.zza Leonardo da Vinci 32, 20133 Milano, Italy

**Keywords:** isatin, multicomponent, oxindole, peptidomimetics, Ugi

## Abstract

An efficient Ugi three-component reaction of a preformed chiral ketimine derived from isatin with various isonitrile and acid components has been developed. The reactions proceeded smoothly and in a stereocontrolled manner with regard to the new center of the Ugi products due to the stereoinduction of the amine chiral residue. A wide variety of novel chiral 3,3-disubstituted 3-aminooxindoles were obtained, a selection of which were subjected to post-Ugi transformations, paving the way to application as peptidomimetics.

## Introduction

**Figure 1:**
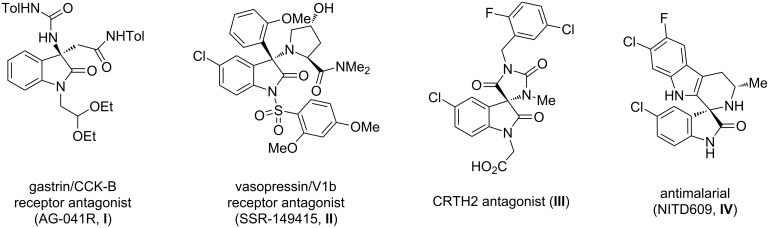
Biologically active agents containing a 3-substituted-3-aminooxindole core.

Isatin and its derivatives have drawn considerable and renewed interest due to their peculiar chemistry and wide range of bioactivities. This led to the development of stereoselective methodologies and the synthesis of compounds with various biological properties [1]. In particular, the high reactivity of the C-3 prochiral carbonyl group allows the easy transformation of isatin into 2-oxindole derivatives, mostly by nucleophilic additions or spiroannulation [2–3]. Oxindoles represent a common structural element in various natural products and biologically active compounds. Diverse oxindole derivatives act as non-peptide scaffolds [4] in peptidomimetic chemistry, either as enzyme inhibitors or as ligands of G-protein-coupled receptors [5]. In particular, 3,3-disubstituted 3-amino-2-oxindoles are present in several drug candidates. They exhibit various types of bioactivity, such as the potent gastrin/CCK-B receptor antagonist **I** [6], the vasopressin VIb receptor antagonist **II** [7–8], the CRTH2 (DP2) receptor antagonist spirohydantoin **III** [9], and the new antimalarial lead **IV** [10–11] (Figure 1).

Giving the importance of this structural motif, the development of rapid synthetic methods for oxindoles bearing a nitrogen atom at the C3-stereogenic center is highly required [12–15]. In the course of our studies on new methodologies to access chiral 3,3-disubstituted 3-aminooxindoles [16–19], we looked at isocyanide-based multicomponent reactions as a possible efficient tool to quickly prepare oxindole-based peptidomimetic compounds [20–22]. Despite the synthetic efficiency of the Ugi reaction and its wide application in combinatorial and medicinal chemistry [23–28], to the best of our knowledge a synthesis of 3,3-disubstituted 3-aminooxindoles which relies on isocyanide-based multicomponent reactions has not been unexplored yet.

Although in this kind of reaction a new stereogenic center is created, the stereoselectivity remains a difficult task. Waiting for enantioselective versions of the Ugi reaction [29–31], the diastereoselective approach by using chiral material is a possible solution. In particular, amines have shown to be promising as chiral auxiliaries, even though only a few examples with satisfactory selectivity were achieved to date [32–36].

## Results and Discussion

Relying on our previous experience, we selected chiral ketimine **1** as a suitable substrate and started to investigate the Ugi three-component reaction (3CR) with *tert*-butyl isocyanide (**2a**) and trifluoroacetic acid (TFA, **3a**) under various reaction conditions. Compound **1** was easily prepared by treatment of isatin with (*S*)-phenyl ethylamine in the presence of MgSO_4_ [16]. The results of this initial study are reported in Table 1.

**Table 1:** Optimization of reaction conditions for the U-3CR of **1** with *tert*-butyl isocyanide and TFA.

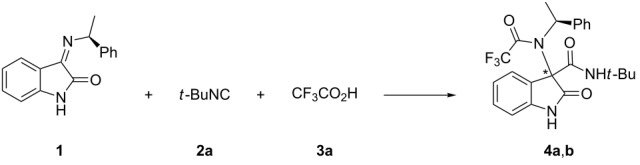						

entry	solvent	time	**1** (0.1 M):**2a**:**3a**	temp.	yield^a^	dr (**a**:**b**)^b^

1	TFE	4 d	1:2:2	rt	complex mixture	–
2	CH_2_Cl_2_	4 d	1:2:2	rt	29	57:43
3	CH_2_Cl_2_	4 d	1:2:3	rt	38	62:38
4	CH_2_Cl_2_	4 d	1:3:3	rt	32	60:40
5	CH_2_Cl_2_	6 d	1:2:3	rt	50	60:40
6	MeOH	4 d	1:2:3	rt	65	85:15
7	MeOH	48 h	1:2:2	rt	77^c^	89:11^c^
8^d^	MeOH	48 h	1:2:2	rt	73	81:19
9^e^	MeOH	48 h	1:2:2	rt	53^c^	90:10^c^
10	MeOH	36 h	1:2:2	65 °C	67	85:15
11^f^	MeOH	3 min	1:2:2	65 °C	69	87:13
12^f^	MeOH	3 min	1:2:2	100 °C	67	71:29
13^f^	neat	3 min	1:2:2	65 °C	37	60:40
14^g^	MeOH	6 h	1:2:2	rt	54	89:11

^a^Isolated yield (%) after chromatographic purification (sum of the two diastereoisomers **a** (major) and **b** (minor)). ^b^dr as determined by ^1^H NMR analysis of the crude. ^c^Average value resulting from two runs. ^d^LiCl (1 equiv) was added. ^e^MgBr_2_ (1 equiv) was added. ^f^Under microwave irradiation (300 W). ^g^Under sonication.

Running the reaction in 2,2,2-trifluoroethanol for 4 days produced only a complex mixture (Table 1, entry 1), while the use of dichloromethane as a solvent allowed us to obtain the desired Ugi product **4** under various reagents ratio albeit in moderate yields and low dr (Table 1, entries 2–5). A change of the solvent to methanol resulted in a pronounced improvement of the reaction. The reaction of imine **1** with 2 equiv of both isocyanide and TFA in methanol (Table 1, entry 7) afforded the product **4** in 77% yield and 89:11 dr after 48 hours at room temperature. A variation of the concentration of **1** in the range from 0.05 M to 0.2 M did not entail any modification, neither in terms of yield nor dr. The remaining imine and a small amount of isatin could be detected from a ^1^H NMR analysis of the crude. The presence of either excess of **3a** (Table 1, entry 6) or additives (Table 1, entry 8 and entry 9) did not affect the results significantly. Surprisingly, the yield decreased upon adding MgBr_2_, even though this promoter was shown to perform well in other reactions on ketimine **1**. Evidently, in this case, the effective protonation of the nitrogen would be required in order to efficiently carry out the attack of the moderately nucleophilic isonitrile. An increase of the reaction temperature (Table 1, entry 10) facilitated a slightly shorter reaction time. Based on this result, we decided to investigate the use of microwave irradiation, with the aim of further increasing the speed of the reaction. Running the reaction in methanol under microwave irradiation (300 W) at 65 °C afforded the product after 3 min, with a yield and a dr almost comparable to that obtained under conventional heating conditions (Table 1, entry 11). An increase of the temperature to 100 °C (Table 1, entry 12) or carrying out the reaction in the absence of a solvent (Table 1, entry 13) led to a reduction of both the yield and the reaction rate. The application of sonication to shorten the reaction times was also useful, since compound **4** was obtained after 6 hours with the same dr, although in a lower yield (Table 1, entry 14).

To exploit the potential of the Ugi reaction to introduce molecular diversity by a one-pot operation, we attempted to realize a four-component (4CR) version. An equimolar mixture of isatin and (*S*)-phenyl ethylamine was reacted with **2a** (2 equiv) and **3a** (2 equiv) in methanol in the presence of MgSO_4_ as a dehydrating agent to promote the formation of the ketimine. Unfortunately, after 4 days, the Ugi product was only afforded in very low yield.

In order to explain the observed diastereoselectivity, the major diastereoisomer **4a**, easily separated from the minor diastereoisomers **4b** by column chromatography, was crystallized from a 1:1 acetone/water solution.

**Figure 2:**
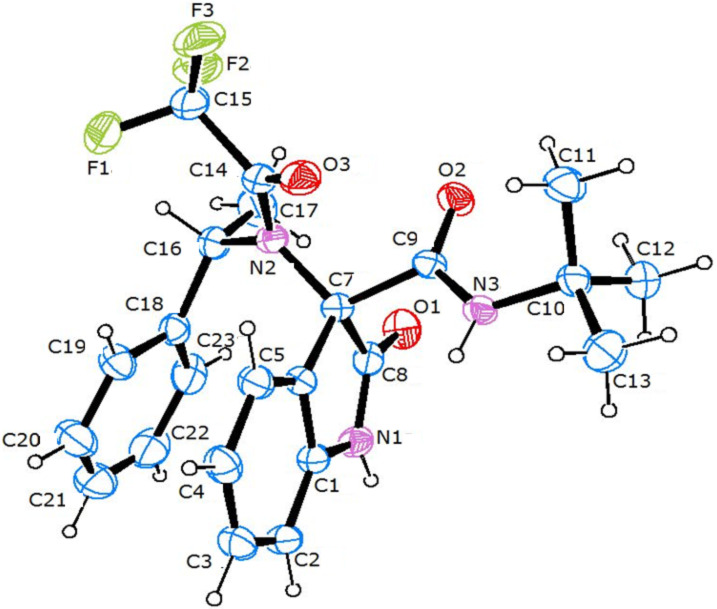
ORTEP [37] view of one of the two independent molecules of **4a** present in the asymmetric unit, showing the arbitrary atom-labelling scheme. Atomic displacement parameters for non-H atoms are at a probability level of 50%.

The X-ray diffraction of the obtained crystal established the absolute configuration *S* at the tetrasubstituted stereocenter C3, which corresponds to C7a of the arbitrary atom-numbering scheme used (Figure 2). The stereochemical assignment *S* was attributed on the basis of the known (*S*)-configuration of the phenyl ethylamine residue.

**Figure 3:**
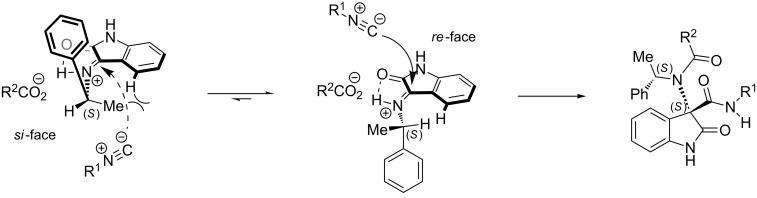
Proposed explanation of the stereochemical outcome of the Ugi 3CR.

This outcome corroborated our previous achievements on addition reactions to ketimine **1**. Figure 3 shows a working model able to explain the observed diastereoselectivity (R^1^ = *t*-Bu, R^2^ = CF_3_). The stereochemical outcome can be justified by the presence of the 1,3-allylic strain, which favors the conformation on the right side of Figure 3. The major diastereoisomer originates from the prevailing delivery of the nucleophile from the less hindered *re* face of the imine double bond.

Aimed to investigate the synthetic scope and limitations of our approach, we finally choose the reaction conditions reported in Table 1, entry 7 as the most convenient ones. Thus, imine **1** was reacted with different isocyanide (**2a–d**) and acid (**3a–g**) components (Table 2). The dr of the products (**4–15**) was determined by a ^1^H NMR analysis of the crude mixture. However, in most cases chromatographic purification allowed the separation of the two diastereoisomers, the major **a** and the minor **b**.

**Table 2:** Reaction of imine **1** with different isocyanide and acid components.^a^

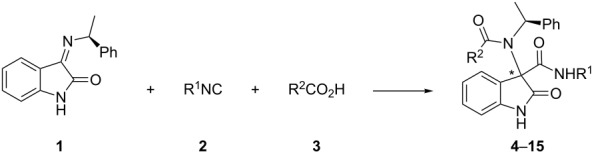					

entry	**2** (R^1^)	**3** (R^2^CO_2_H)	yield^b^	dr (**a**:**b**)^c^	compd

1	**2a** (*t-*Bu)	**3a** (TFA)	77	89:11	**4a**,**b**
2	**2a** (*t-*Bu)	**3b** (HCO_2_H)	62	63:37	**5a**,**b**
3	**2b** (CH_2_CO_2_Me)	**3a** (TFA)	70	88:12	**6a**,**b**
4	**2b** (CH_2_CO_2_Me)	**3b** (HCO_2_H)	66	70:30	**7a**,**b**
5	**2c** (Bn)	**3a** (TFA)	66	65:35	**8a**,**b**
6	**2c** (Bn)	**3b** (HCO_2_H)	70	64:36	**9a**,**b**
7	**2d** [C(CH_3_)_2_CH_2_(CH_3_)_3_]	**3b** (HCO_2_H)	48	69:31	**10a**,**b**
8	**2a** (*t-*Bu)	**3c** (*N*-Boc-L-Ala-OH)	51	62:38	**11a**,**b**
9	**2a** (*t-*Bu)	**3d** (*N*-Boc-L-Pro-OH)	31	77:23	**12a**,**b**
10	**2a** (*t-*Bu)	**3e** (*N*-Boc-D-Pro-OH)	18	96:4	**13a**,**b**
11	**2a** (*t-*Bu)	**3f** (*N*-Ac-Gly-OH)	complex mixt.	–	
12	**2a** (*t-*Bu)	**3g** (mono*-*ethyl fumarate)	74	62:38	**14a**,**b**
13	**2c** (Bn)	**3g** (mono*-*ethyl fumarate)	47	59:41	**15a**,**b**

^a^Reaction conditions: **1** (0.1 M), **2** (2 equiv), **3** (2 equiv), MeOH, rt, 48 h. ^b^Isolated yield (%) after chromatographic purification (sum of the two diastereoisomers **a** (major) and **b** (minor)). ^c^dr as determined by ^1^H NMR analysis of the crude.

Most of the products were obtained in acceptable yields, while the dr proved to be more dependent on varying the different components of the reaction. An exchange of the acid TFA with formic acid (Table 2, entries 2, 4, 6 and 7) resulted in a decrease of the dr. This may be caused by a minor stereofacial differentiation of the intermediate carboxylate iminium ion during the nucleophilic addition of the isocyanide. Isocyanide **2b** (Table 2, entry 3 and entry 4), formally derived from glycine, afforded satisfactory results, thus letting us foresee the application of this approach to isocyanides prepared from natural amino acids. If compared with *tert*-butylisocyanide (**2a**), benzylisocyanide (**2c**) (Table 2, entries 5, 6 and 13) seems to be less effective in terms of an acceptable dr, while the yields were still good. Of interest was also the product **10**, derived from a reaction of isocyanide **2d** (Table 2, entry 7), since it can be easily converted into the correspondent primary amide derivative (vide infra).

To explore potential applications of the Ugi reaction products in the field of peptidomimetics, we also tested the reaction of ketimine **1** in the presence of different *N*-Boc-protected amino acids as acid components. The use of L-amino acids **3c** and **3d** (Table 2, entry 8 and entry 9) afforded the desired products with moderate yields. The best result in terms of de (54%) was achieved with the more hindered proline. The reaction of the glycine derivative **3f** produced a complex inseparable mixture of products (Table 2, entry 11). When *N*-Boc-D-Pro was used as the acid component (Table 2, entry 10) a high 96:4 dr was measured despite a low yield (18%). This outcome suggests a matching/mismatching effect between the chirality of ketimine **1** and that of the D-Pro or L-Pro reagent, respectively. Satisfactory yields were also achieved with mono-ethyl fumarate as the acid component (Table 2, entry 12 and entry 13). The obtained products **14** and **15** can be further elaborated to yield more complex structures (vide infra).

**Scheme 1:**
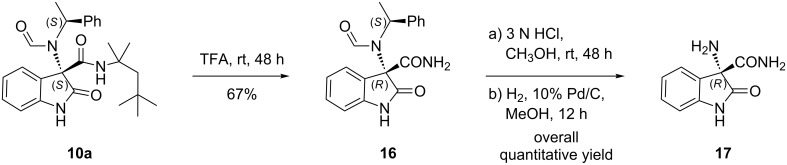
Post-Ugi transformation on compound **10a**.

Next, selected post-Ugi transformations were investigated in order to better evaluate the synthetic versatility of the Ugi adducts. Compound **10a** was easily converted to the primary amide **16** and then to the known amino amide **17** [16], thus establishing the possible subsequent functionalization of both the primary amine and the isonitrile-derived primary carboxamide functional groups (Scheme 1). Also, this chemical correlation of the major diastereoisomer **10a** allowed us to further confirm the prevailing *S*-configuration at the tetrasubstituted stereocenter C3 of the Ugi products.

**Scheme 2:**
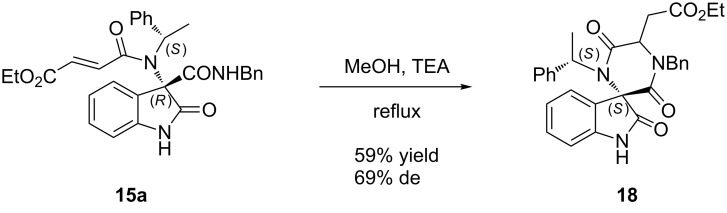
Post-Ugi cyclization on compound **15a**.

Compound **15a** was submitted to a post-Ugi cyclization, namely an intramolecular aza-Michael [24] reaction, which afforded compound **18** bearing the privileged spiro-diketopiperazine scaffold (Scheme 2). Spiro-diketopiperazines are present in many natural products [38–40] and have recently received much attention as pharmacologically active peptidomimetics [41–43].

The reaction proceeded smoothly in methanol under reflux in the presence of excess TEA to give the product **18** by a regioselective six-exo-trig cyclization.

## Conclusion

We have developed a novel approach to the synthesis of optically active 3,3-disubstituted 3-aminooxindoles by means of a three-component Ugi reaction. A number of compounds could be smoothly obtained in satisfactory yields (up to 77%) with various levels of diastereoselectivity (up to 96:4 dr). The synthetic versatility of the Ugi adducts was demonstrated by applications of post-Ugi transformations. Importantly, this represents the first example where an isocyanide-based multicomponent reaction has been applied to an isatin-derived ketimine, thus highlighting the promising reactivity of this derivative as a precursor of chiral 3,3-disubstituted 3-aminooxindoles.

## Supporting Information

Supporting information features the experimental section, crystallographic data, general methods and copies of NMR spectra (^1^H and ^13^C) for all new compounds.

File 1 Experimental section and crystallographic data.

File 2 General methods and copies of NMR spectra for all new compounds.
