# Supplementary material for: Asymmetric Ugi 3CR on isatin-derived ketimine: synthesis of chiral 3,3-disubstituted 3-aminooxindole derivatives
Source: Beilstein J Org Chem. 2014 Jun 18;10:1383–9. doi: 10.3762/bjoc.10.141 (PMC4077467; doi:10.3762/bjoc.10.141)
Supplement: File 1 — Experimental section and crystallographic data. [file Beilstein_J_Org_Chem-10-1383-s001.pdf]

Supporting Information File 1

for

Asymmetric Ugi 3CR on isatin-derived ketimine: synthesis of chiral 3,3-disubstituted 3-aminooxindole derivatives

Giordano Lesma[1], Fiorella Meneghetti[2], Alessandro Sacchetti[3], Mattia Stucchi[1], Alessandra Silvani*[1]

Address: [1]Dipartimento di Chimica, Università degli Studi di Milano, via C. Golgi, 19, 20133 Milano (Italy), [2]Dipartimento di Scienze Farmaceutiche, Università degli Studi di Milano, via L. Mangiagalli 25, 20133 Milano, Italy and [3]Dipartimento di Chimica, Materiali e Ingegneria Chimica 'Giulio Natta', Politecnico di Milano, p.zza Leonardo da Vinci 32, 20133 Milano, Italy

Email: Alessandra Silvani - alessandra.silvani@unimi.it

*Corresponding author

Experimental section and crystallographic data

Table of Contents

Experimental section S2
Crystallographic data S7
References S7

Experimental section

General procedure for the Ugi 3CR on 1

To a 0.10 M solution of the known ketimine **1** [1] (0.40 mmol, 1 equiv) in CH_3OH, the appropriate isocyanide (2.0 equiv) and the acid (2.0 equiv) were added at room temperature. The solution was stirred at rt for 48 h, then the mixture was concentrated in vacuo. The crude was then purified by flash chromatography on silica gel column using *n*-hexane/EtOAc (2:1 to 1:3) as the eluent, to give products **4–15**. Isolated yields (%) after chromatographic purification are reported in Table 1 and Table 2 together with diastereoisomeric ratios (dr) as determined on the crude. The following analytical data refer to the major diasereoisomer **a**, if not otherwise stated.

(*S*)-*N*-*tert*-Butyl 2-oxo-3-(2,2,2-trifluoro-*N*-((*S*)-1-phenylethyl)acetamido)indoline-3-carboxamide (4a). White foam; R_f = 0.60 (*n*-hexane-EtOAc, 1:2); $[\alpha]_D^{20}$= + 222.0 (*c* 0.93, $CHCl_3$). ^{1}H NMR (300 MHz, $CDCl_3$) δ 7.49 (d, *J*= 7.7 Hz, 1H), 7.18 (t, *J*= 7.8 Hz, 1H), 7.17-6.94 (m, 6H), 6.80 (br, s, 1H), 6.46 (d, *J* = 7.8 Hz, 1H), 5.45 (q, *J* = 7.0Hz, 1H), 5.43 (br, m, 1H), 2.13 (d, *J*= 7.0 Hz, 3H), 1.14 (s, 9H); ^{13}C NMR (75 MHz, $CDCl_3$) δ 170.8, 162.3, 159.0 (q, *J*= 39.2 Hz), 140.6, 135.8, 130, 128.8 (2C), 127.9, 127.6 (2C), 127.3, 126.8, 123.6, 116.8 (q, *J*= 287.8 Hz), 110.0, 73.8, 56.9, 51.8, 28.6 (3C), 18.4; HRMS (EI) calculated for $C_{23}H_{24}F_3N_3O_3$ 447.1770 found 447.1776.

(*S*)-*N*-*tert*-Butyl 2-oxo-3-(*N*-((*S*)-1-phenylethyl)formamido)indoline-3-carboxamide (5a). White amorphous solid; R_f = 0.42 (*n*-hexane-EtOAc, 1:2). $[\alpha]_D^{20}$= - 134.3 (*c* 0.50, $CHCl_3$). ^{1}H NMR (300 MHz,$CDCl_3$) δ 8.33 (s, 1H), 8.22 (br, s, 1H), 7.44-7.30 (m, 4H), 7.23 (m, 2H), 7.15 (t, *J* = 7.8 Hz, 1H), 7.04 (d, *J* = 7.6 Hz, 1H), 6.93 (t, *J* = 7.6 Hz, 1H), 6.71 (d, *J* = 7.7 Hz, 1H), 4.98 (q, *J*= 7.1 Hz, 1H), 1.85 (d, *J*= 7.1 Hz, 3H), 1.37 (s, 9H). ^{13}C NMR (75 MHz, $CDCl_3$) δ 175.0, 163.2, 161.1, 142.5, 141.1,129.5, 128.9 (2C), 127.6, 126.4, 126.1 (2C), 123.4, 123.0, 110.5, 71.1, 55.5, 52.0, 27.8 (3C), 24.7. HRMS (EI) calculated for $C_{22}H_{25}N_3O_3$ 379.1896 found 379.1889.

Methyl 2-(2-oxo-3-(2,2,2-trifluoro-*N*-((*S*)-1-phenylethyl)acetamido)indoline-3-carboxamido)acetate (6a and 6b). As an unseparable mixture of two diastereoisomers (0.88:0.12 ratio): Foam; R_f = 0.49 (*n*-hexane-EtOAc, 2:1). ^{1}H NMR (400 MHz, $CDCl_3$) δ 8.24-8.20 (m, 2H), 7.48 (d, *J* = 7.5 Hz, 1H), 7.35 (t, *J* = 7.5 Hz, 1H), 7.23 (t, *J*= 7.6 Hz, 1H), 7.19-6.97 (m, 5H), 6.83 (d, *J* = 7.7 Hz, 1H), 5.55 (m, 0.88H), 5.00 (q, *J* = 7.0 Hz, 0.12H), 4.31 (d, *J*= 17.5 Hz, 0.88H), 4.22 (d, *J* = 17.5 Hz, 0.88H), 3.86- 3.75 (m, 0.24H), 3.77 (s, 0.36H), 3.66 (s, 2.64H), 2.18 (br, d, *J* = 7.0 Hz, 2.64H), 1.41 (d, *J* = 7.0 Hz, 0.36H). ^{13}C NMR (100 MHz, $CDCl_3$) δ 176.5, 169.0, 168.7, 159.0 (q, *J*= 35.4 Hz), 142.4 and 142.1 (1C), 141.6 and 141.0 (1C), 131.3, 130.3 (2C), 129.1, 128.6 (2C), 127.0 and 126.9 (1C), 126.3, 124.5 and 124.3 (1C), 117.3 (q, *J*= 288.2 Hz), 111.9 and 111.0 (1C), 72.8, 57.4 and 54.2 (1C), 53.4 and 53.0 (1C), 43.0 and 42.6 (1C), 21.1 and 19.2 (1C). HRMS (EI) calculated for $C_{22}H_{20}F_3N_3O_5$ 463.1355 found 463.1361.

(*R*)-Methyl 2-(2-oxo-3-(*N*-((*S*)-1-phenylethyl)formamido)indoline-3-carboxamido)acetate (7a). Pale yellow oil; R_f = 0.30 (*n*-hexane-EtOAc, 1:2); $[\alpha]_D^{20}$= - 14.5 (*c* 1.17, $CHCl_3$); ^{1}H NMR (300 MHz, $CDCl_3$) δ 8.37 (br, s, 1H), 8.23 (s, 1H), 7.50 (br, d, *J* = 7.8 Hz, 1 H), 7.44-7.13 (m, 6H), 6.93 (t, *J* = 7.6 Hz, 1 H), 6.78 (d, *J* = 7.8 Hz, 1H), 6.23 (br, m, 1H), 5.09 (br, q, *J*= 7.2 Hz, 1H), 4.15 (dd, *J* = 18.5 and 5.9 Hz, 1H), 3.89 dd, *J* = 18.5 and 5.9 Hz, 1H), 3.73 (s, 3H), 1.80 (d, *J* = 7.2 Hz, 3H); ^{13}C NMR (75 MHz, $CDCl_3$) δ 174.0, 169.7, 163.6, 161.4, 141.3, 141.2, 129.9, 128.8 (2C), 127.6, 127.2 (2C), 126.6, 125.6, 123.2, 110.5, 71.2, 56.5, 52.5, 39.8, 23.1; HRMS (EI) calculated for $C_{21}H_{21}N_3O_5$ 395.1481 found 395.1491

(*R*)-*N*-Benzyl 2-oxo-3-(2,2,2-trifluoro-*N*-((*S*)-1-phenylethyl)acetamido)indoline-3-carboxamide (8a). Oil; R_f = 0.54 (*n*-hexane-EtOAc, 2:1); $[\alpha]_D^{25}$ = + 208.4 (*c* 0.94, $CHCl_3$); ^{1}H NMR (300 MHz, DMSO-d_6, 80 °C) δ 7.41-7.17 (m, 10H), 7.12-6.96 (m, 4H), 6.86 (t, *J* = 7.8 Hz, 1H), 6.63 (br, d, *J* = 7.7 Hz, 1H), 4.82 (q, *J* = 7.4 Hz, 1H), 4.23 (dd, *J* = 14.6 and 5.9 Hz, 1H), 4.04 (dd, *J* = 14.6 and 4.9 Hz, 1H), 1.92 (d, *J* = 7.4 Hz, 3H); ^{13}C NMR (75 MHz, $CDCl_3$) δ 177.9, 172.1, 164.4 (q, *J*= 34.6 Hz), 140.7, 140.4, 136.5, 130.1, 128.6-127.3 (11C), 124.2, 123.7, 116.7 (q, *J*= 287.8 Hz), 110.1, 72.5, 56.6, 44.1, 18.6; HRMS (EI) calculated for $C_{26}H_{22}F_3N_3O_3$ 481.1613 found 481.1601.

(*R*)-*N*-Benzyl 2-oxo-3-(*N*-((*S*)-1-phenylethyl)formamido)indoline-3-carboxamide (9a). Amorphous solid; R_f = 0.35 (*n*-hexane-EtOAc, 1:2); $[\alpha]_D^{22}$ = - 127.0 (*c* 1.08, $CHCl_3$); ^{1}H NMR (300 MHz,$CDCl_3$) δ 8.30 (s, 1H), 8.14 (s, 1H), 7.61 (d, *J* = 7.7 Hz, 1H), 7.41-7.03 (m, 12H), 6.97-6.94 (m, 1H), 6.74 (d, *J*= 7.7 Hz, 1H), 5.05 (q, *J* = 7.0 Hz, 1H), 4.28 (dd, *J* = 14.7, 5.5 Hz, 1H), 3.95 (dd, *J* = 14.7, 5.5 Hz, 1H), 1.84 (d, *J* = 7.0 Hz, 3H). ^{13}C NMR (75 MHz, $CDCl_3$) δ 174.4, 162.9, 162.8, 141.9, 141.0, 136.6, 129.8, 128.8-126.2 (11C), 125.8, 123.7, 110.5, 70.5, 56.3, 44.3, 24.2; HRMS (EI) calculated for $C_{25}H_{23}N_3O_3$ 413.1739 found 413.1752.

***N*-(2,4,4-Trimethylpentan-2-yl) (*S*)-2-Oxo-3-(*N*-((*S*)-1-phenylethyl)formamido)indoline-3-carboxamide (10a)**. Foam; R_f = 0.80 (*n*-hexane-EtOAc, 1:2); $[\alpha]_D^{18}$= + 75.0 (*c* 0.97, $CHCl_3$); ^{1}H NMR (400 MHz, $CDCl_3$) δ 8.40 (s, 1H), 8.36 (s, 1H), 7.47 (d, *J* = 7.5 Hz, 2H), 7.41 (t, *J* = 7.6 Hz, 2H), 7.35 (tt, *J* = 7.6 and 2.5 Hz, 1H), 7.17 (td, *J* = 7.6 and 1.5 Hz, 1H), 7.11-705 (br, m, 2H), 6.95 (td, *J* = 7.6 and 1.5 Hz, 1H), 6.73 (d, *J* = 7.7 Hz, 1H), 5.02 (q, *J*= 7.1 Hz, 1H), 1.85 (d, *J* = 7.1 Hz, 3H), 1.49 (s, 3H), 1.47 (s, 3H), 0.91 (s, 9H), 0.90 (s, 2H); ^{13}C NMR (100 MHz, $CDCl_3$) δ 175.5, 163.5, 161.9, 142.0, 141.4, 130.0, 129.6 (2C), 128.8, 127.8 (2C), 127.6, 126.9, 123.7, 110.8, 72.5, 56.9, 56.8, 52.7, 32.1, 31.9 (3C), 29.1, 28.8, 24.0. HRMS (EI) calculated for $C_{26}H_{33}N_3O_3$ 435.2522 found 435.2519.

(*S*)-*tert*-Butyl ((2*S*)-1-((3-(*tert*-butylcarbamoyl)-2-oxoindolin-3-yl)((*S*)-1-phenylethyl)amino)-1-oxopropan-2-yl)carbamate (11a). Oil; R_f = 0.59 (*n*-hexane-EtOAc, 1:2); $[\alpha]_D^{25}$= + 51.9 (*c* 1.07, $CHCl_3$); ^{1}H NMR (400 MHz, $CDCl_3$) δ 8.18 (s, 1H), 7.78 (d, *J* = 7.7 Hz, 2H), 7.65 (d, *J* = 7.5 Hz,

1H), 7.42 (t, *J* = 7.7 Hz, 2H), 7.35 (tt, *J* = 7.7 and 2.1 Hz, 1H), 7.26 (t, *J* = 7.6 Hz, 1H), 7.09 (t, *J* = 7.6 Hz, 1H), 6.81 (d, *J* = 7.7 Hz, 1H), 6.64 (br, m, 1H), 5.16-5.06 (m, 2H), 4.47 (m, 1H), 1.89 (d, *J* = 7.1 Hz, 3H), 1.43 (s, 9H), 1.32 (s, 9H), 0.56 (d, *J* = 6.5 Hz, 3H); ^{13}C NMR (100 MHz, $CDCl_3$) δ 177.3, 171.5, 162.8, 154.9, 140.8, 140.4, 129.4, 128.6 (2C), 128.0, 127.9, 127.8, 126.8, 126.1, 123.4, 110.1, 79.5, 75.0, 56.0, 52.0, 48.8, 28.3 (3C), 28.2 (3C), 19.0, 18.5; HRMS (EI) calculated for $C_{29}H_{38}N_4O_5$ 522.2842 found 522.2855.

(*S*)-*tert*-Butyl-2-(((*S*)-3-(*tert*-butylcarbamoyl)-2-oxoindolin-3-yl)((*S*)-1-phenylethyl)carbamoyl)pyrrolidine-1-carboxylate (12a). Amorphous foam; R_f = 0.57 (*n*-hexane-EtOAc, 1:2); $[\alpha]_D^{30}$ = + 46.2 (*c* 0.93, $CHCl_3$); ^{1}H NMR (400 MHz, $CDCl_3$, 0.1:0.3:0.3:0.3 rotameric mixture) δ 8.61 (s, 0.1H), 8.52 (s, 0.3H), 8.49 (s, 0.3H), 8.43 (s, 0.3H), 7.83-6.68 (m, 9.9H), 6.11 (br, m 0.1H), 5.19-4.98 (br, m, 0.6H), 4.53 (br, dd, *J* = 8.1 and 2.5 Hz, 0.1H), 4.44 (m, 0.3H), 4.36 (dd, *J* = 8.5 and 2.3 Hz, 0.3H), 4.32 (d, *J* = 8.2 Hz, 0.3H), 3.61-3.18 (m, 2.4H), 2.37-1.72 (m, 4H), 1.84 (br, d, *J* = 5.6 Hz, 3H), 1.52-1.43 (m, 9H), 1.35-1.24 (m, 9H); ^{13}C NMR, (100 MHz, $CDCl_3$) δ 172.9 and 172.8 (1C), 165.4 and 164.7 (1C), 155.1 and 154.6 (1C), 151.8 and 150.1 (1C), 141.5 (2C), 131.5-110.8 (10C), 81.3 and 79.9 (2C), 76.2 and 76.1 (1C), 60.2-56.0 (2C), 48.3-47.0 (1C), 31.5-29.7 (1C), 29.4-28.8 (6C), 25.1-24.1 (1C), 20.2-18.9 (1C); HRMS (EI) calculated for $C_{31}H_{40}N_4O_5$ 548.2999 found 548.2995.

(*R*)-*tert*-Butyl 2-(((*S*)-3-(*tert*-butylcarbamoyl)-2-oxoindolin-3-yl)((*S*)-1-phenylethyl)carbamoyl)pyrrolidine-1-carboxylate (13a). Amorphous white solid; R_f = 0.63 (*n*-hexane-EtOAc, 2:1); $[\alpha]_D^{19}$= -25.5 (*c* 0.82, $CHCl_3$); ^{1}H NMR (400 MHz, DMSO-d_6, 100°C) δ 10.33 (br, m, 1H), 7.63 (d, *J* = 7.0 Hz, 2H), 7.54 (d, *J*= 7.6 Hz, 1H), 7.31 (t, *J* = 7.6 Hz, 2H), 7.28-7.18 (m, 2H), 6.99 (t, *J* = 7.6 Hz, 1H), 6.82 (d, *J* = 7.6 Hz, 1H), 5.90 (br, m, 1H), 5.29 (q, *J* = 7.3 Hz, 1H), 4.34 (t, *J* = 5.2 Hz, 1H), 3.41–3.33 (m, 1H), 3.32–3.24 (m, 1H), 1.86-1.76 (br, m, 3H), 1.73-1.61 (m, 1H), 1.59 (d, *J* = 7.3 Hz, 3H), 1.40 (s, 9H), 1.14 (s, 9H); ^{13}C NMR, (75 MHz, DMSO-d_6, rotameric mixture) δ 176.3 and 175.4 (1C), 1173.5 and 172.7 (1C), 163.8 (1C), 153.2 and 152.6 (1C), 142.9 and 142.3 and 142.0 and 141.4 (2C), 131.3-109.6 (10C), 79.2, 78.6, 73.8 and 73.2 (1C), 57.8 and 57.4 and 57.0 and 56.0 and 55.5 (2C), 50.7 and 48.4 (1C), 30.8 and 30.2 (1C), 28.5-27.9 (6C), 24.0-22.5 (1C), 20.6-19.0 (1C); HRMS (EI) calculated for $C_{31}H_{40}N_4O_5$ 548.2999 found 548.3005.

(*E*)-Ethyl 4-(((*S*)-3-(*tert*-butylcarbamoyl)-2-oxoindolin-3-yl)((*S*)-1-phenylethyl)amino)-4-oxobut-2-enoate (14a). Amorphous white solid; R_f = 0.66 (*n*-hexane-EtOAc, 1:2). $[\alpha]_D^{22}$ = + 91.9 (*c* 0.75, $CHCl_3$); ^{1}H NMR (400 MHz, $CDCl_3$) δ 8.54 (s, 1H), 7.65 (br, m, 1H), 7.57 (d, *J* = 7.8 Hz, 2H), 7.40 (t, *J* = 7.7 Hz, 2H), 7.31 (br, t, *J* = 7.5 Hz, 1H), 7.26 (td, *J* = 7.7 and 1.2 Hz, 1H), 7.1 (t, *J* = 7.7 Hz, 1H), 6.91 (br, m, 1H), 6,87 (br, d, *J* = 15.1 Hz, 1H), 6.82 (d, *J* = 7.7 Hz, 1H), 6.55 (d, *J*= 15.1 Hz, 1H), 5.21 (q, *J* = 7.0 Hz, 1H), 4.15-4.07 (m, 2H), 1.91 (d, *J* = 7.0 Hz, 3H), 1.34 (s, 9H),

1.21 (t, *J* = 7.1 Hz, 3H); ^{13}C NMR (100 MHz,$CDCl_3$) δ 175.1, 166.9, 165.1, 162.5, 141.1 (2C), 135.4, 131.1, 129.7, 128.8 (2C), 127.6, 127.0, 126.9, 126.7 (2C), 123.2, 110.5, 74.5, 60.9, 56.0, 52.2, 28.2 (3C), 21.1, 14.0; HRMS (EI) calculated for $C_{27}H_{31}N_3O_5$ 477.2264 found 477.2259.

(*E*)-Ethyl 4-(((*S*)-3-(benzylcarbamoyl)-2-oxoindolin-3-yl)((*S*)-1-phenylethyl)amino)-4-oxobut-2-enoate (15a). Thick oil; R_f = 0.58 (*n*-hexane-EtOAc, 1:2); $[\alpha]_D^{24}$ = + 51.5 (*c* 1.00, $CHCl_3$); ^{1}H NMR (400 MHz, $CDCl_3$) δ 8.50 (s, 1H), 7.72 (d, *J* = 7.7 Hz, 1H), 7.56 (d, *J* = 7.8 Hz, 2H), 7.44-7.17 (m, 9H), 7.07 (t, *J* = 7.7 Hz, 1H), 6.94 (br, m, 1H), 6.88 (d, *J* = 15.4 Hz, 1H), 6.79 (d, *J* = 7.7 Hz, 1H), 6.57 (d, *J* = 15.4 Hz, 1H), 5.07 (br, q, *J* = 7.0 Hz, 1H), 4.44 (dd, *J* = 14.8 and 5.9 Hz, 1H), 4.37 (dd, *J* = 14.8 and 5.6 Hz, 1H), 4.11 (m, 2H), 1.73 (d, *J* = 7.0 Hz, 3H), 1.21 (t, *J* = 7.1 Hz, 3H); ^{13}C NMR (100 MHz, $CDCl_3$) δ 174.5, 167.0, 165.1, 162.7, 141.0, 140.8, 136.7, 135.2, 131.1, 129.9, 128.9-128.6 (4C), 127.8-127.6 (4C), 128.8, 128.4 (2C), 128.1, 123.2, 110.5; 73.9, 61.0, 56.2, 44.2, 20.5, 14.0; HRMS (EI) calculated for $C_{30}H_{29}N_3O_5$ 511.2107 found 511.2116.

(*R*)-2-Oxo-3-(*N*-((*S*)-1-phenylethyl)formamido)indoline-3-carboxamide (16). A round-bottomed flask containing **10a** (47 mg, 0.11 mmol) was put in an ice-cold water bath and trifluoroacetic acid (1 mL) was added. After ten minutes the bath was removed and the mixture was stirred at room temperature for two days. The solvent was evaporated in vacuo and the crude was treated with an aqueous solution of saturated $NaHCO_3$ to pH 8. The aqueous layer was then extracted with EtOAc (3 × 10 mL) and the combined organic extracts were dried over Na_2SO_4, filtered and the solvent was removed under reduced pressure. The obtained crude was purified by silica gel flash chromatography (*n*-hexane/EtOAc 1:9) to give 23 mg (67% yield) of compound **16**, as a light pink oil; R_f = 0.20 (*n*-hexane/EtOAc 3:7); $[\alpha]_D^{18}$ = - 97.4 (*c* 0.38, CH_3OH); ^{1}H NMR (300 MHz, $CDCl_3$, 0.15:0.85 rotameric mixture) δ 9.37 (s, 0.15H), 9.03 (s, 0.85H), 8.50 (s, 0.15H), 8.36 (s, 0.85H), 7.27-7.25 (m, 4H), 7.20-7.14 (m, 3H), 6.93 (t, *J* = 7.7 Hz, 1H), 6.75 (d, *J* = 7.7 Hz, 1H), 6.43-6.13 (br, m, 2H), 5.06 (q, *J* = 7.0 Hz, 0.15H), 4.85 (q, *J* = 7.0 Hz, 0.85H), 1.65 (d, *J* = 7.0 Hz, 2.5H), 1.51 (d, *J*= 7.0 Hz, 0.5H); ^{13}C NMR (75 MHz, $CDCl_3$) δ 175.2 and 173.8 (1C), 168.1 and 166.8 (1C), 163.5 and 162.4 (1C), 141.4, 140.7, 130.1, 128.7 (2C), 128.0, 127.1 (2C), 126.8, 125.5, 123.0, 111.3 and 110.8 (1C), 71.9 and 71.3 (1C), 56.6 and 53.5 (1C), 22.6 and 17.2 (1C); HRMS (EI) calculated for $C_{18}H_{17}N_3O_3$ 323.1270 found 323.1279.

(*R*)-3-Amino-*N*-benzyl-2-oxoindoline-3-carboxamide (17). To compound **16** (28 mg, 0.085 mmol), a solution of 3 N HCl in methanol (1 mL) was added and the resulting mixture was stirred at room temperature for two days. The solution was basified to pH 9 with saturated aqueous $NaHCO_3$ solution, then extracted with EtOAc (3 × 10 mL). The combined organic extracts were dried over Na_2SO_4, filtered and the solvent was removed under reduced pressure, affording 25 mg (quantitative yield) of the known (*R*)-*N*-benzyl-2-oxo-3-(((*S*)-1-phenylethyl)amino)indoline-3-

carboxamide. $[\alpha]_D^{25}$= - 276.2 (*c* 1, CH_3OH) [from literature: $[\alpha]_D^{20}$= - 274.6 (*c* 1, CH_3OH)]. This crude was used without further purification and quantitatively converted to the known compound **17**. $[\alpha]_D^{25}$= - 68.4 (*c* 0.5, CH_3OH) [from literature: $[\alpha]_D^{20}$= - 72.4 (*c* 0.5, CH_3OH)]. Spectroscopic data of **17** are in agreement with the literature [1].

Ethyl 2-((2'*S*)-4'-benzyl-2,3',6'-trioxo-1'-((*S*)-1-phenylethyl)spiro[indoline-3,2'-piperazine]-5'-yl)acetate (18). A solution of **15a** (60 mg, 0.12 mmol) and TEA (167 µL, 1.2 mmol) in anhydrous CH_3OH (3 mL) was heated at reflux for 7 hours and then the solvent was removed in vacuo. The crude was purified by silica gel flash chromatography (*n*-hexane/EtOAc 1:1), to afford compound **18** as an inseparable 1.5:1 mixture of diastereoisomers (59% yield). Wax; R_f = 0.43 (*n*-hexane-EtOAc, 1:2); ^{1}H NMR (400 MHz, $CDCl_3$, mixture of diastereoisomers) δ 8.23 (br, m, 0.6H), 7.67 (s, 0.4H), 7.43-7.26 (m, 7.4H), 7.26-7.14 (m, 2.3H), 7.14-7.01 (m, 2.3H), 6.98 (d, *J* = 7.6 Hz, 1H), 6.90-6.72 (m, 1H), 5.13 (d, *J* = 14.9 Hz, 0.6H), 5.11 (d, *J* = 15.2 Hz, 0.4H), 4.87 (br, t, *J* = 5.5 Hz, 0.4H), 4.68 (br, t, *J* = 5.4 Hz, 0.6H), 4.42 (m, 06H), 4.30 (d, *J* = 15.2 Hz, 0.4H), 4.27 (d, *J* = 14.9 Hz, 0.6H), 4.24-4.03 (m, 2.4H), 3.43 (dd, *J* = 16.6 and 5.5 Hz, 0.4H), 3.23-3.07 (m, 1.6H), 1.63 (d, *J* = 7.0 Hz, 1.2H), 1.60 (d, *J* = 7.0 Hz, 1.8H), 1.29 (br, m, 1.2H), 1.21 (t, *J*= 7.1 Hz, 1.8H); ^{13}C NMR (100 MHz, $CDCl_3$, mixture of diastereoisomers) δ 174.5 and 173.8 (1C), 171.5 and 171.2 (1C), 168.5 and 166.7(1C), 163.2 and 162.9 (1C), 142.6 and 142.4 (1C), 140.3, 136.2 and 135.7 (1C), 131.5 and 131.4 (1C), 129.7-127.8 (10C), 126.5, 126.0 and 125.6 (1C), 124.0, 111.9 and111.7 (1C), 73.0, 62.0 and 61.9 (1C), 59.0 and 58.2 (1C), 57.4, 49.0 and 48.6 (1C), 42.0 and 41.1 (1C), 18.5 and 17.9 (1C), 14.7; HRMS (EI) calculated for $C_{30}H_{29}N_3O_5$ 511.2107 found 511.2114.

Crystallographic data

Crystals of **4a** were obtained by slow evaporation of a 1:1 acetone/water solution at room temperature, as colourless elongated prisms. Diffraction data have been collected by means of a Bruker-Axs CCD-based three circle diffractometer, working at ambient temperature with graphite-monochromatized MoKα X-radiation (λ = 0.71073 Å). Omega-rotation frames (scan width 0.3°, scan time 20 s, sample-to-detector distance 50 mm) were processed with the SAINT software [2] for data reduction (including intensity integration, background, Lorentz and polarization corrections) and for determination of accurate unit-cell dimensions, obtained by least-squares refinement of the positions of 5349 independent reflections with $I > 10\sigma(I)$ in the 2θ range 4–40°. Absorption effects were empirically evaluated by the SADABS software [3] and absorption correction was applied to the data. The structure, which presents two independent molecules and one acetone solvent moiety in the asymmetric unit, was solved by direct methods [4] and the refinement was carried out with SHELX-97 [5] All non-H-atoms were refined anisotropically. The positions of hydrogen atoms were introduced at calculated positions, in their described geometries and allowed to ride on the attached carbon atom with fixed isotropic thermal parameters (1.2 Ueq of the parent carbon atom). CCDC-963823 contains the supplementary crystallographic data for this paper. These data can be obtained free of charge via www.ccdc.cam.ac.uk/conts/retrieving.html (or from the Cambridge Crystallographic Data Centre, 12, Union Road, Cambridge CB21EZ, UK; fax: ++44 1223 336 033; or deposit@ccdc.cam.ac.uk).

Crystal data for 4a: $C_{24.5}H_{27}F_3N_3O_{3.5}$, M_r = 476.49 g/mol, Monoclinic, Space group $P2_1$, a = 9.4623(8) Å, b = 11.2493(9) Å, c = 23.160(2) Å, β = 96.046(2)°, V = 2451.5(4) Å^3, Z = 4, D_{calc} = 1.291 Mg/m^3, R = 0.049 (32834 reflections/8515 unique), $wR2$ = 0.126, T = 293(2)K, GOF = 1.018. The reflections were collected in the range $0.9° \leq \theta \leq 25.0°$ employing a 0.60 × 0.13 × 0.08 crystal.

References

1. Sacchetti, A.; Silvani, A.; Gatti, F. G.; Lesma, G.; Pilati, T.; Trucchi, B. *Org. Biomol. Chem.* **2011,** *9*, 5515–5522.
2. Bruker: SAINT Software Reference Manual. Version 6, Bruker AXS Inc., Madison, Wisconsin, USA, 2003.
3. Sheldrick, G. M. SADABS Siemens Area Detector Absorption Correction Program, University of Göttingen, Göttingen, Germany, 1996.
4. Altomare, A.; Cascarano, G.; Giacovazzo, C.; Guagliardi, A.; Burla, M. C.; Polidori, G.; Camalli, M. *J. Appl. Cryst*., **1994**, *27*, 435.
5. Sheldrick, G. M. *Acta Cryst*. **2008**, A64, 112-122.
