# Supplementary material for: Asymmetric Ugi 3CR on isatin-derived ketimine: synthesis of chiral 3,3-disubstituted 3-aminooxindole derivatives
Source: Beilstein J Org Chem. 2014 Jun 18;10:1383–9. doi: 10.3762/bjoc.10.141 (PMC4077467; doi:10.3762/bjoc.10.141)

Supporting Information File 2

for

Asymmetric Ugi 3CR on isatin-derived ketimine: synthesis of chiral 3,3-disubstituted 3-aminooxindole derivatives

Giordano Lesma[1], Fiorella Meneghetti[2], Alessandro Sacchetti[3], Mattia Stucchi[1], Alessandra Silvani*[1]

Address: [1]Dipartimento di Chimica, Università degli Studi di Milano, via C. Golgi, 19, 20133 Milano (Italy), [2]Dipartimento di Scienze Farmaceutiche, Università degli Studi di Milano, via L. Mangiagalli 25, 20133 Milano, Italy and [3]Dipartimento di Chimica, Materiali e Ingegneria Chimica 'Giulio Natta', Politecnico di Milano, p.zza Leonardo da Vinci 32, 20133 Milano, Italy

Email: Alessandra Silvani - alessandra.silvani@unimi.it

*Corresponding author

General methods and copies of NMR spectra for all new compounds

Table of Contents

General methods S2

^{1}H NMR and ^{13}C NMR (APT) spectra of compounds **4–18** S3

General methods

All solvents were distilled and properly dried, if necessary, prior to use. All chemicals were purchased from commercial sources and used directly, unless indicated otherwise. All reactions were run under N_2, unless otherwise indicated. All reactions were monitored by thin layer chromatography (TLC) on precoated silica gel 60 F254; spots were visualized with UV light or by treatment with 1% aqueous $KMnO_4$ solution. Products were purified by flash chromatography on silica gel 60 (230–400 mesh).

NMR spectra were recorded with 400 MHz spectrometers. Chemical shifts (δ) are expressed in ppm relative to TMS at δ = 0 ppm for ^{1}H NMR and relative to $CDCl_3$ at δ = 77.16 ppm for ^{13}C NMR. ^{13}C NMR spectra have been recorded using the APT pulse sequence; the signals of CH and CH_3 are positive while CH_2 and quarternary carbons are negative. High-resolution mass spectra were recorded with FT-ICR (Fourier Transform Ion Cyclotron Resonance) instruments, equipped with ESI or EI source.

Compound **4a**: ^{1}H NMR (300 MHz, $CDCl_3$)

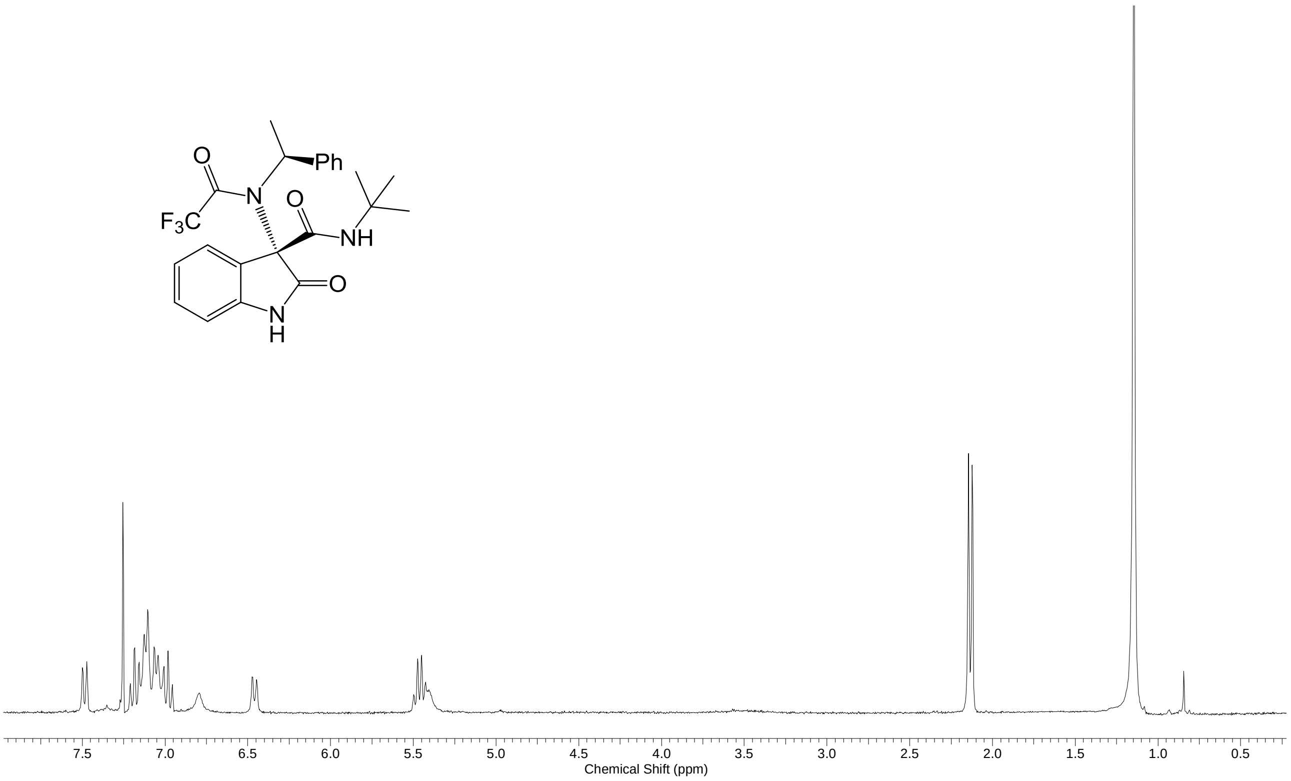

Compound **4a**: ^{13}C NMR (75 MHz, $CDCl_3$)

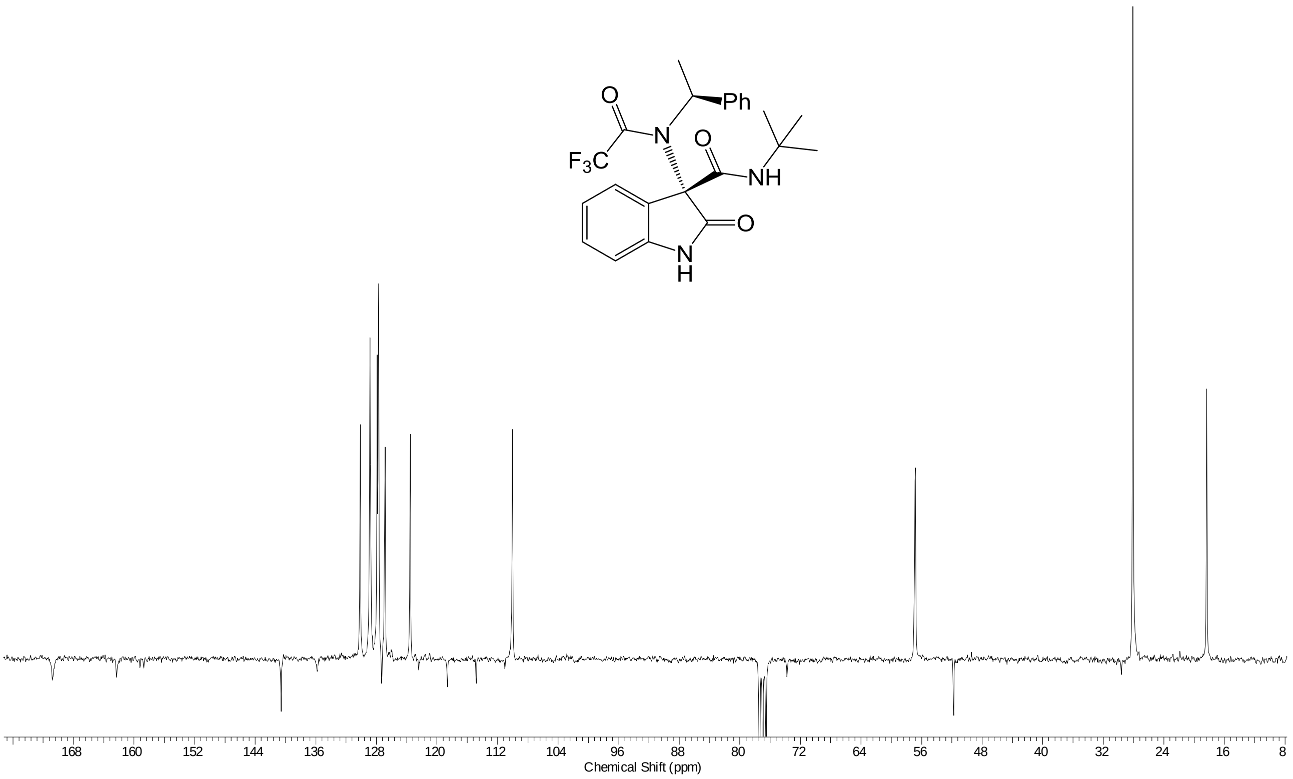

Compound **5a**: ^{1}H NMR (300 MHz, $CDCl_3$)

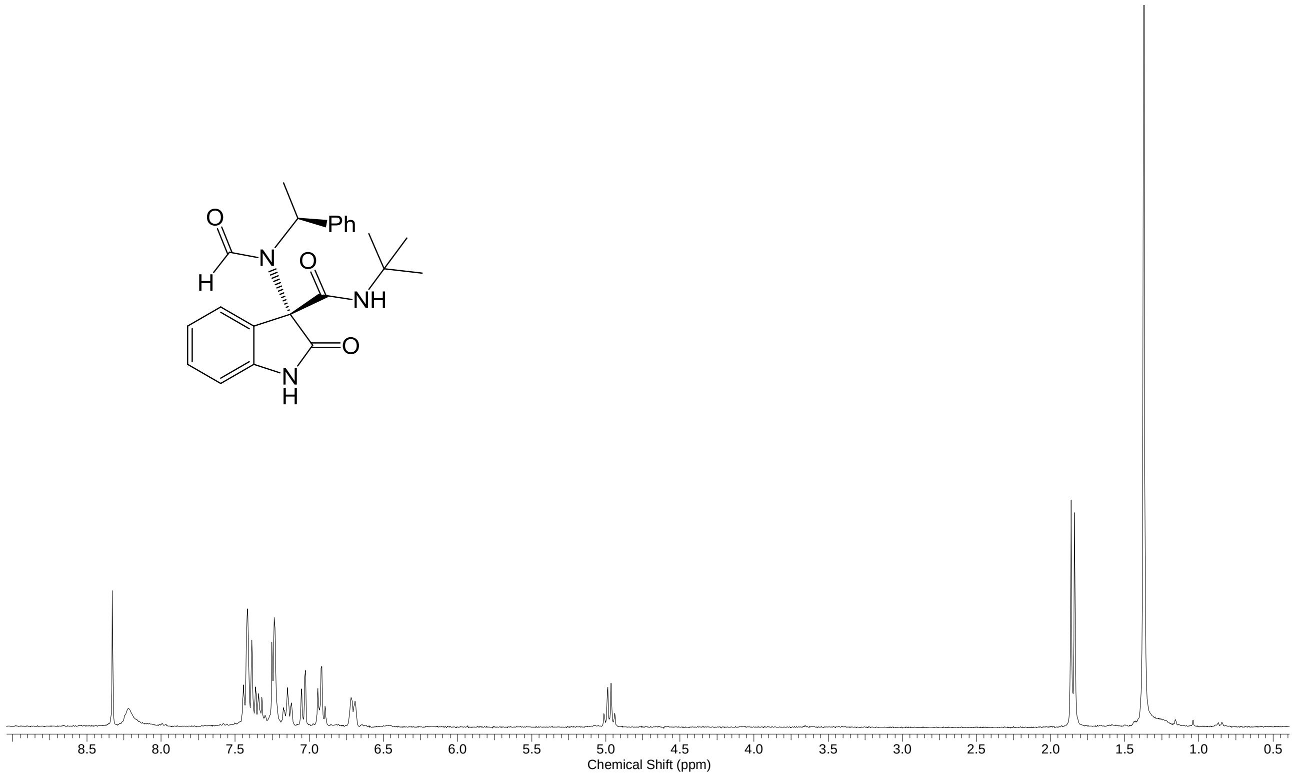

Compound **5a**: ^{13}C NMR (75 MHz, $CDCl_3$)

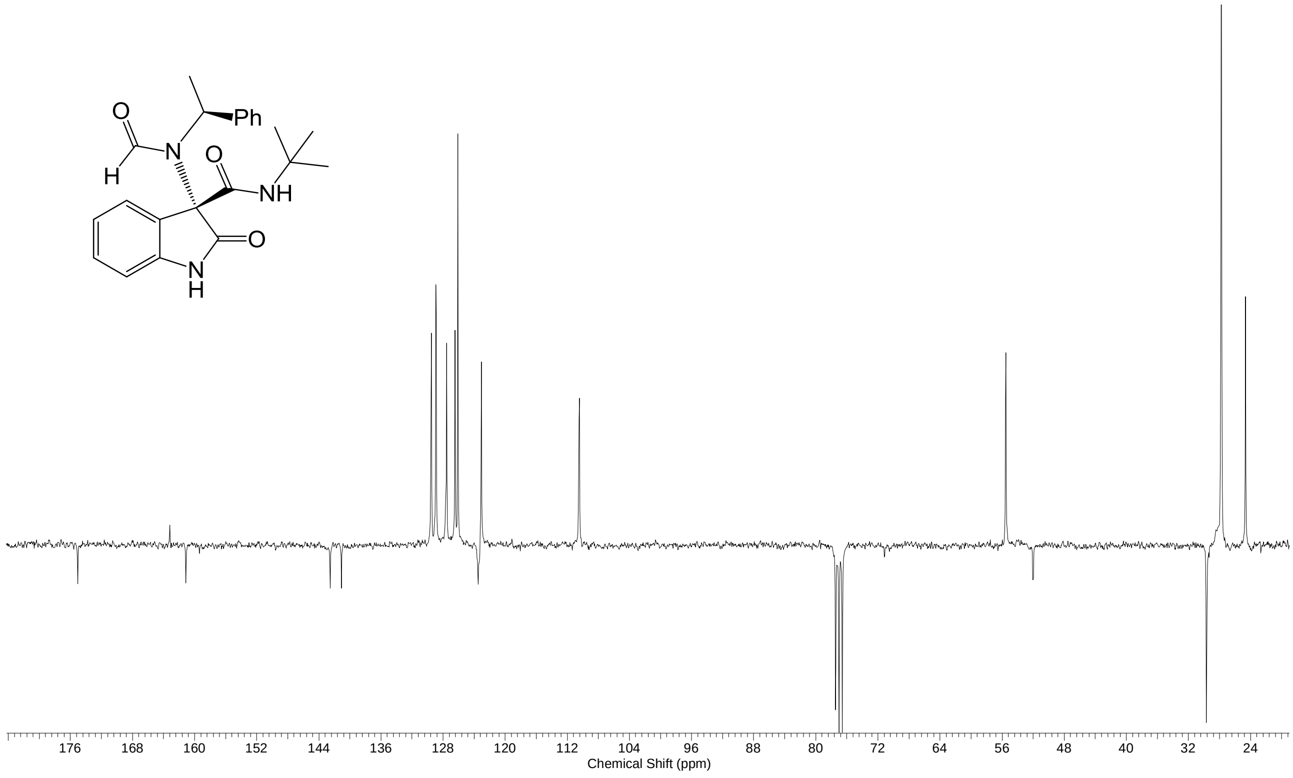

Compound **6**: ^{1}H NMR (400 MHz, $CDCl_3$, diastereoisomeric mixture)

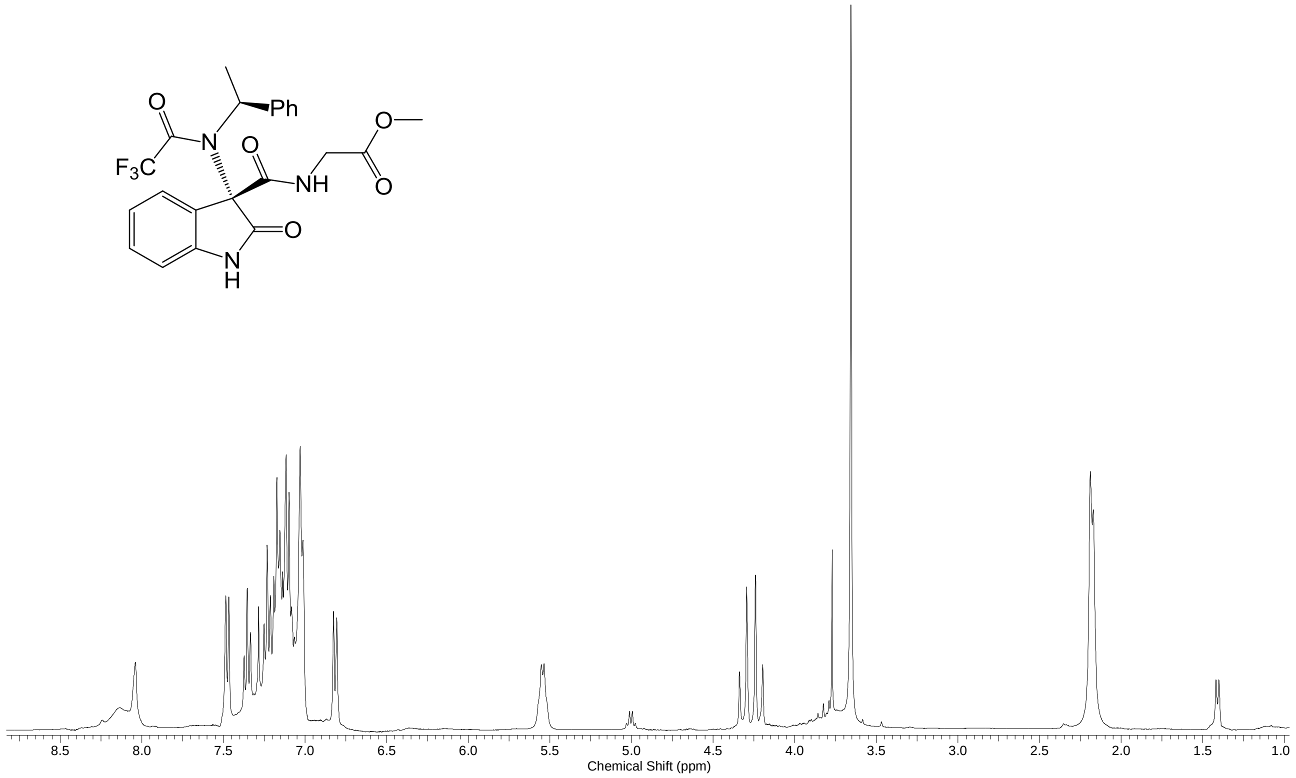

Compound **6**: ^{13}C NMR (100 MHz, $CDCl_3$, diastereoisomeric mixture)

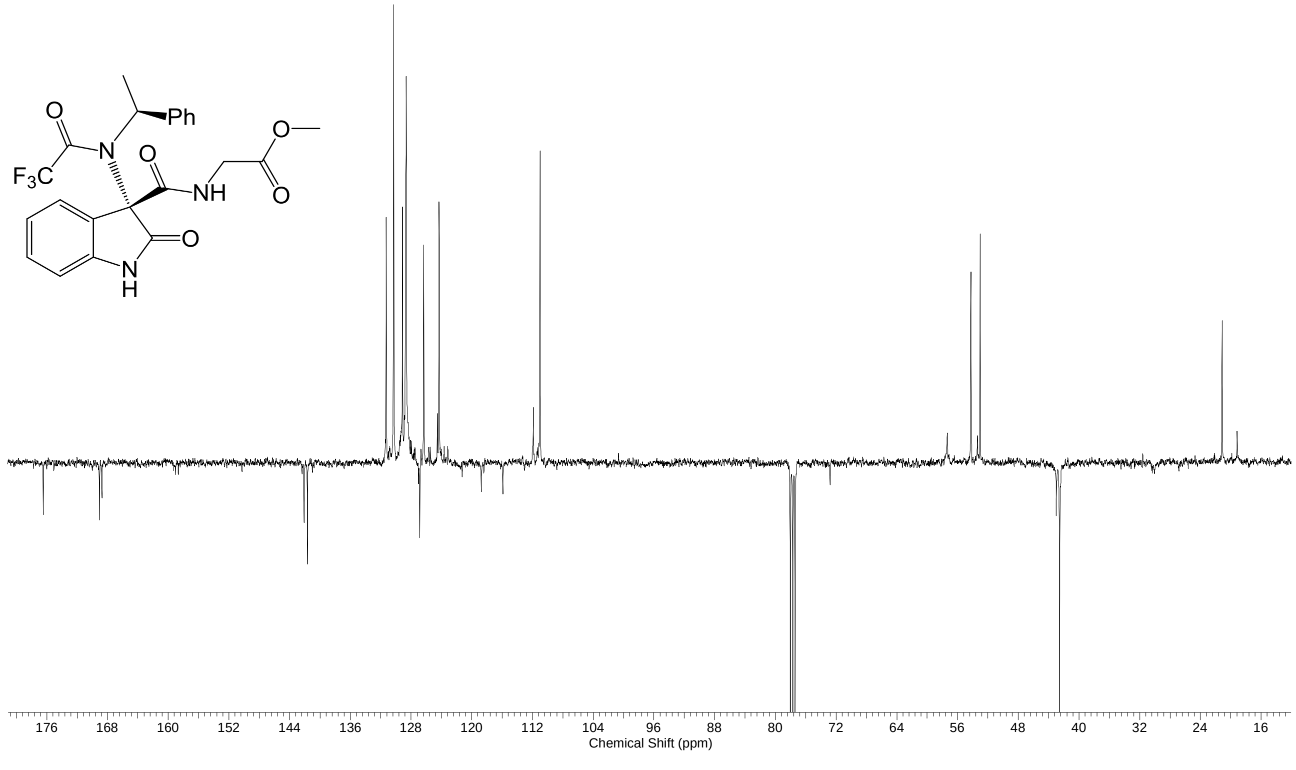

Compound **7a**: ^{1}H NMR (300 MHz, $CDCl_3$)

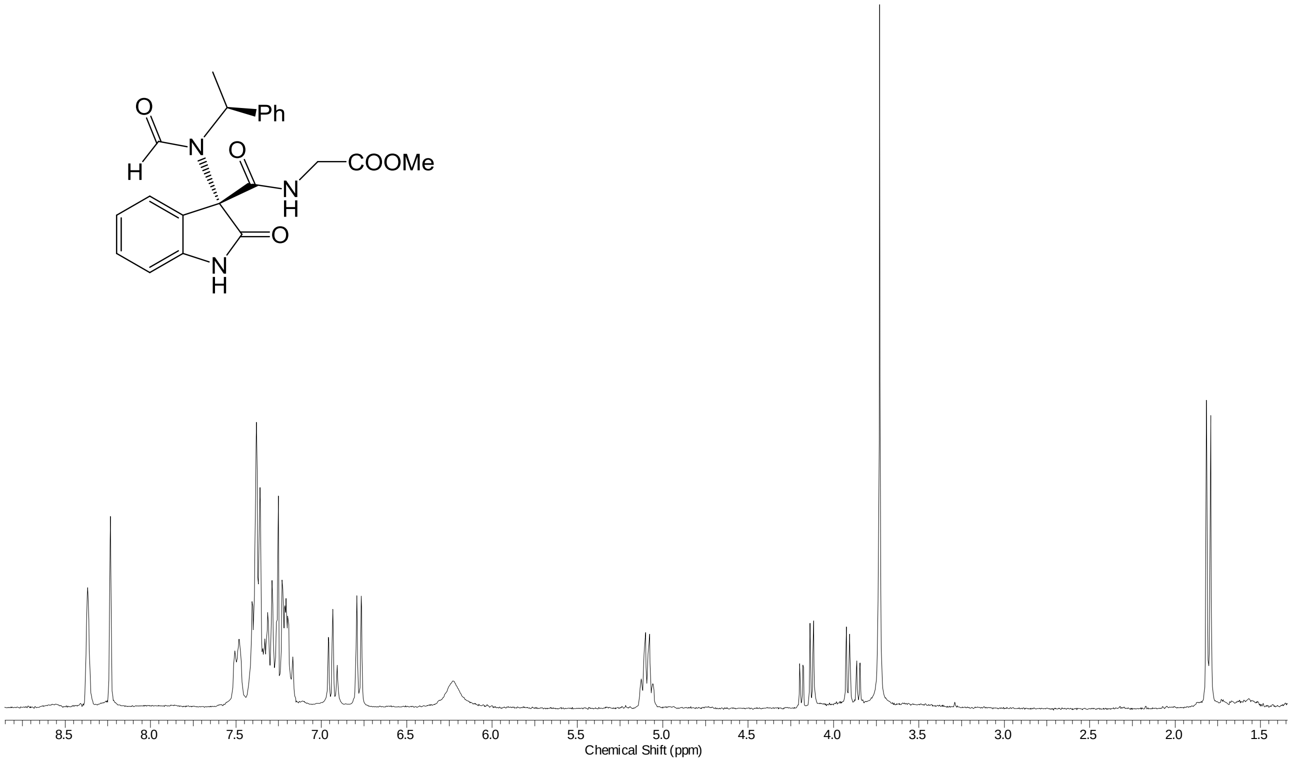

Compound **7a**: ^{13}C NMR (75 MHz, $CDCl_3$)

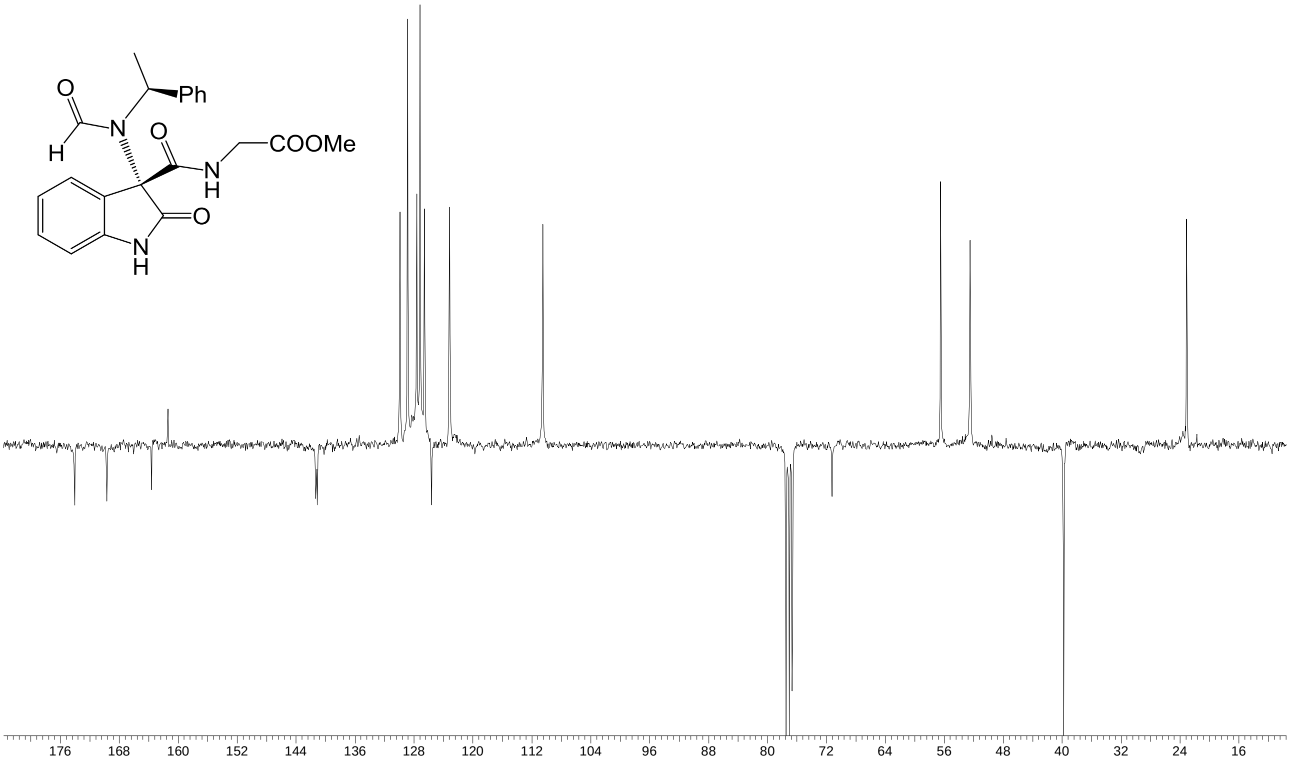

Compound **8a**: ^{1}H NMR (300 MHz, DMSO d_6, 80 °C)

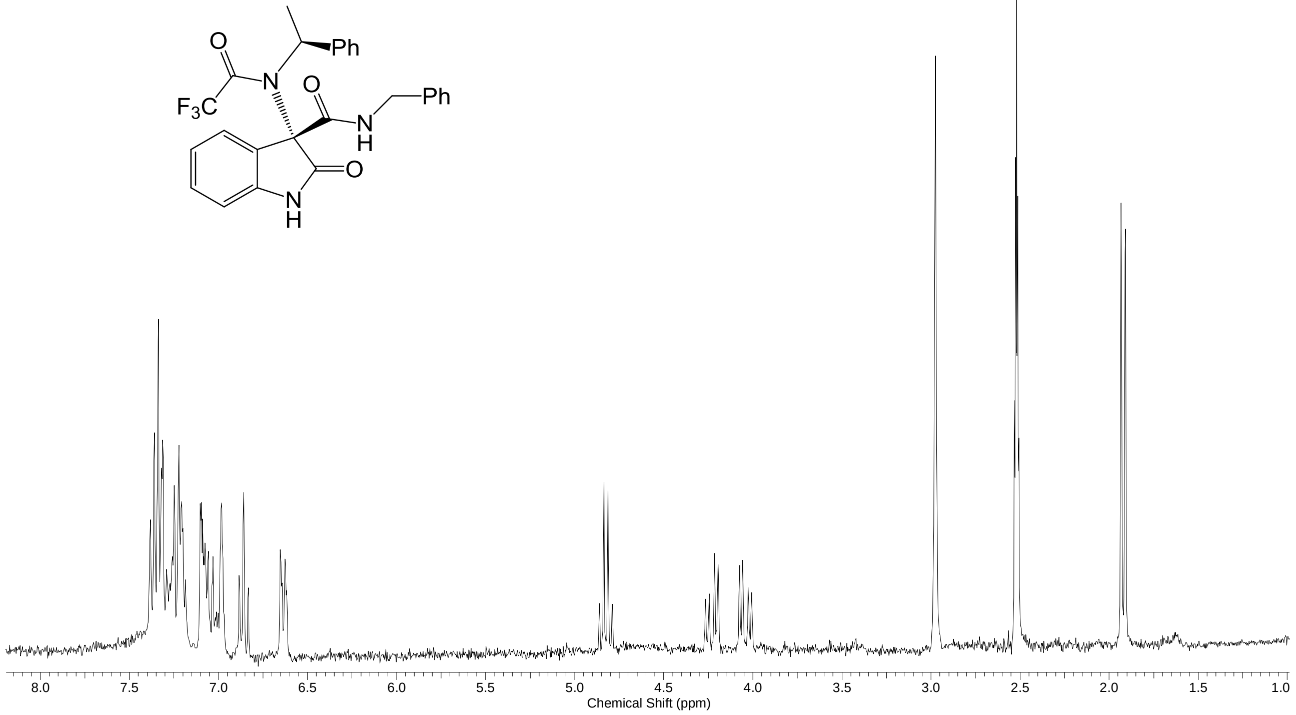

Compound **8a**: ^{13}C NMR (75 MHz, $CDCl_3$)

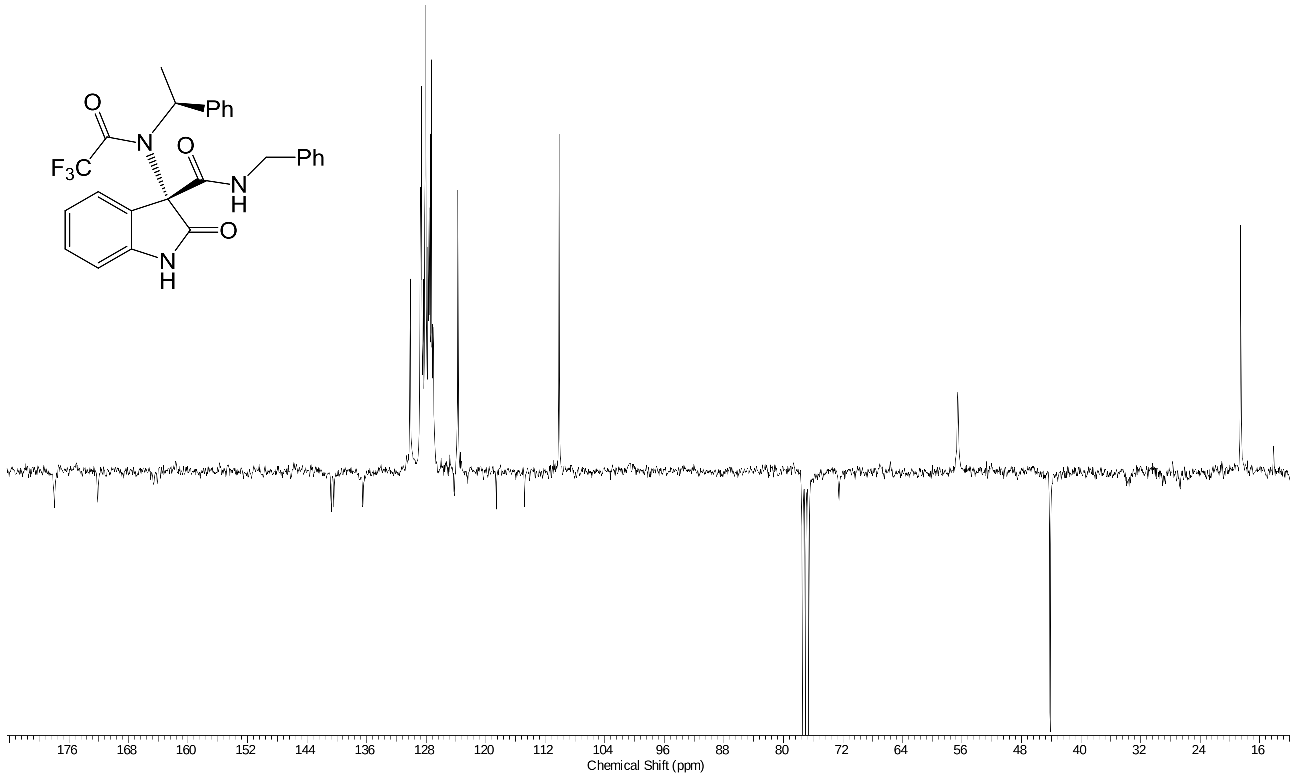

Compound **9a**: ^{1}H NMR (300 MHz, $CDCl_3$)

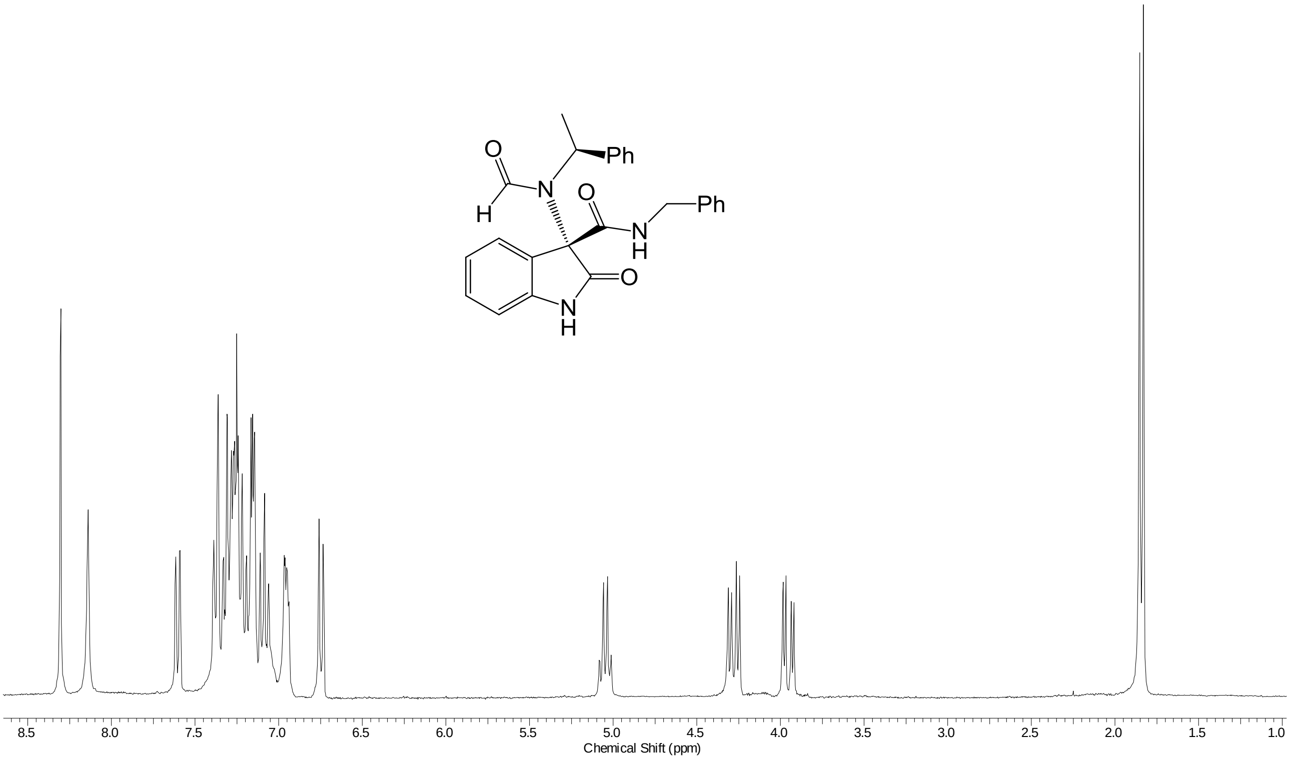

Compound **9a**: ^{13}C NMR (75 MHz, $CDCl_3$)

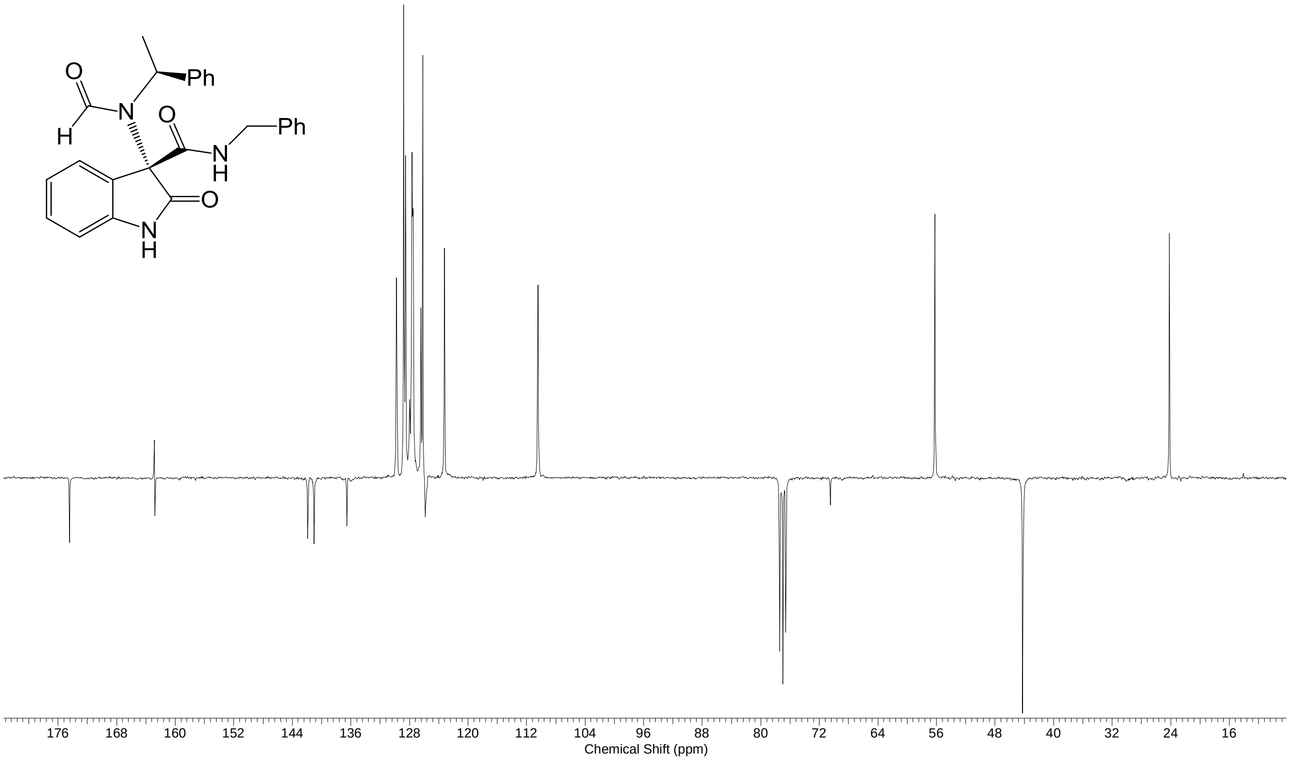

Compound **10a**: ^{1}H NMR (400 MHz, $CDCl_3$)

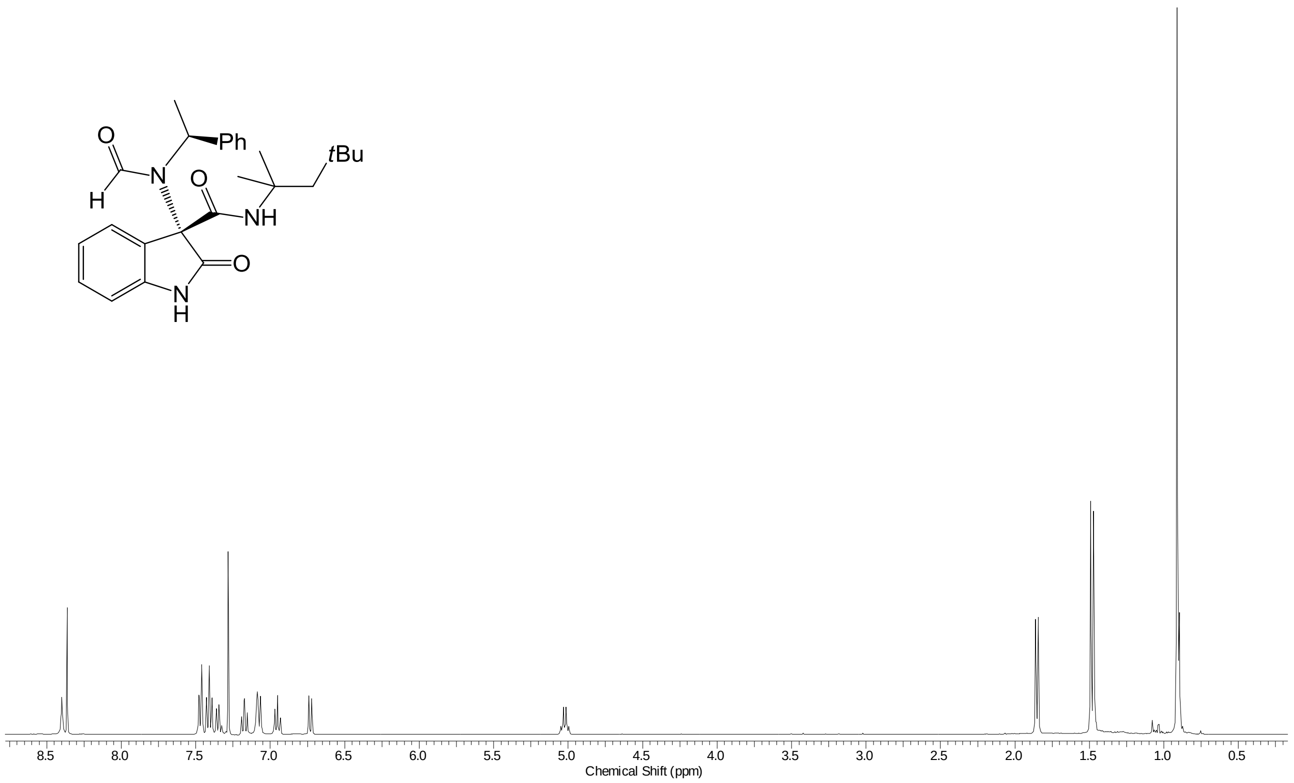

Compound **10a**: ^{13}C NMR (100 MHz, $CDCl_3$)

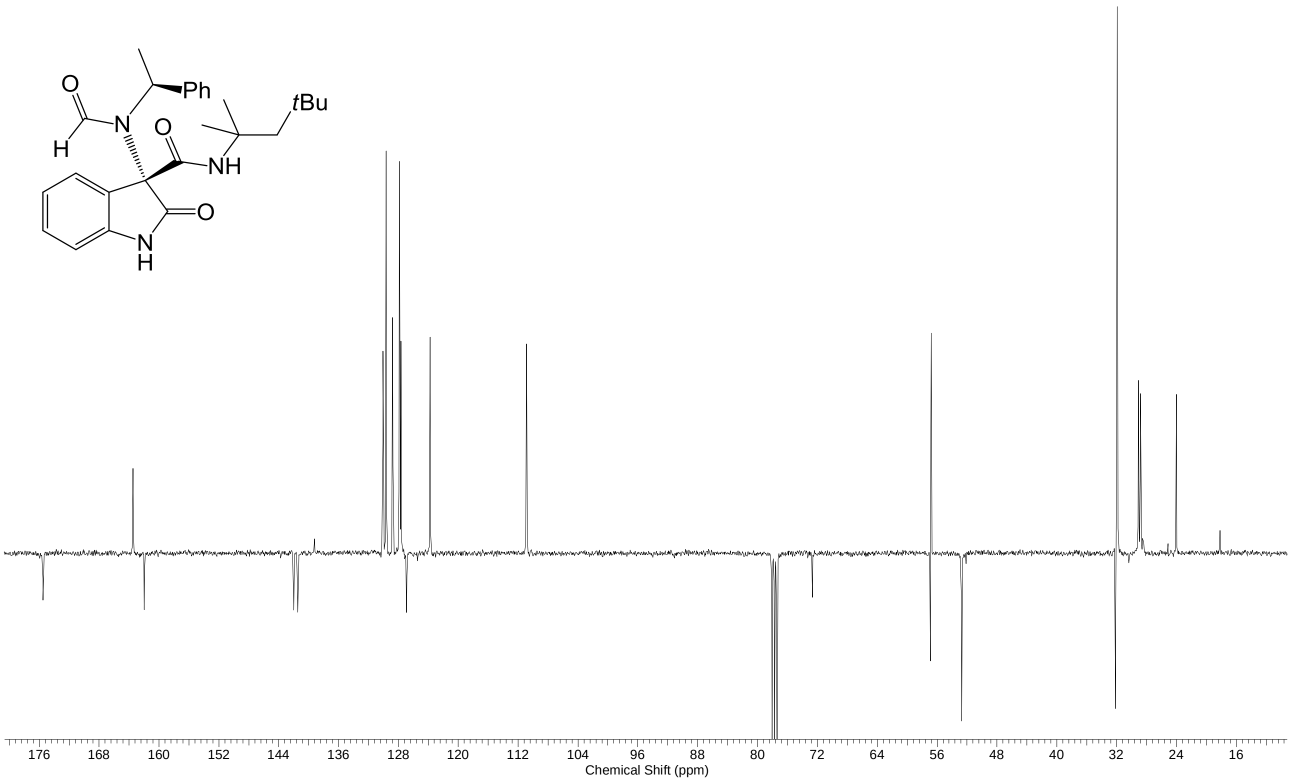

Compound **11a**: ^{1}H NMR (400 MHz, $CDCl_3$)

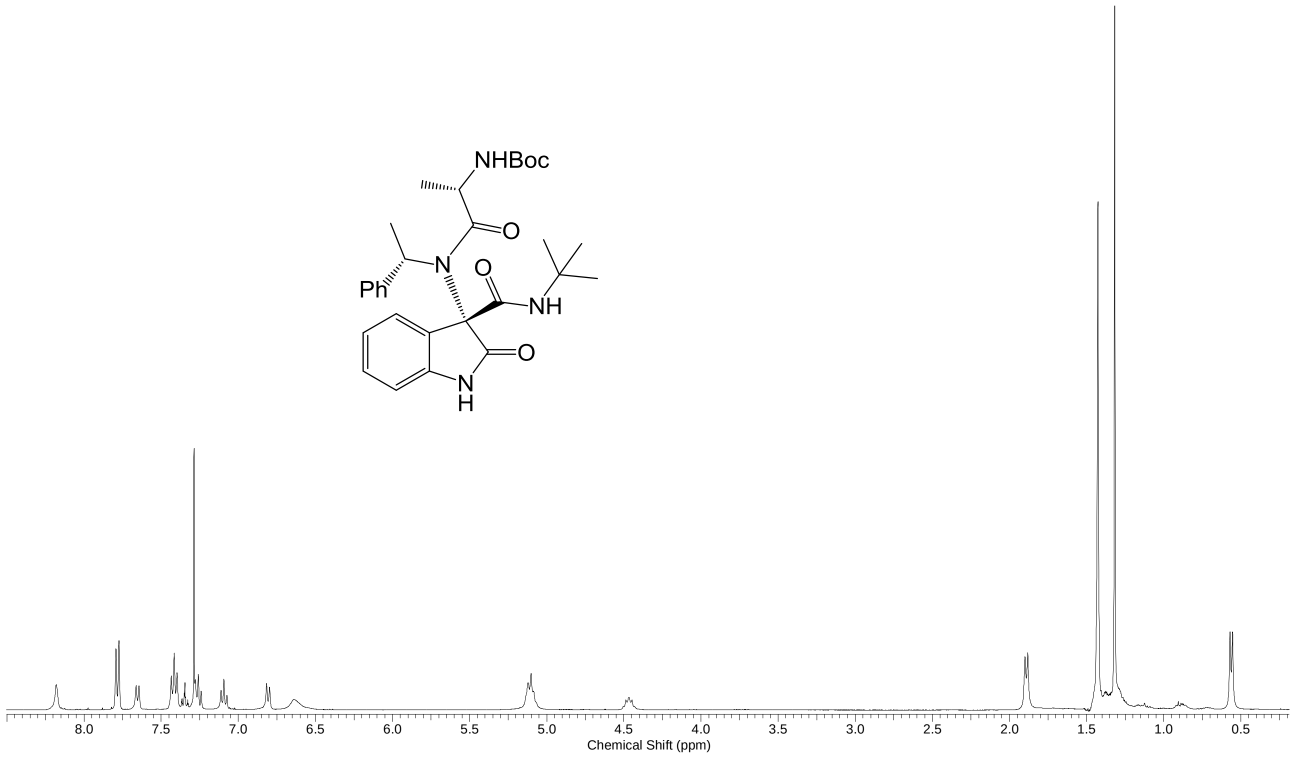

Compound **11a**: ^{13}C NMR (100 MHz, $CDCl_3$)

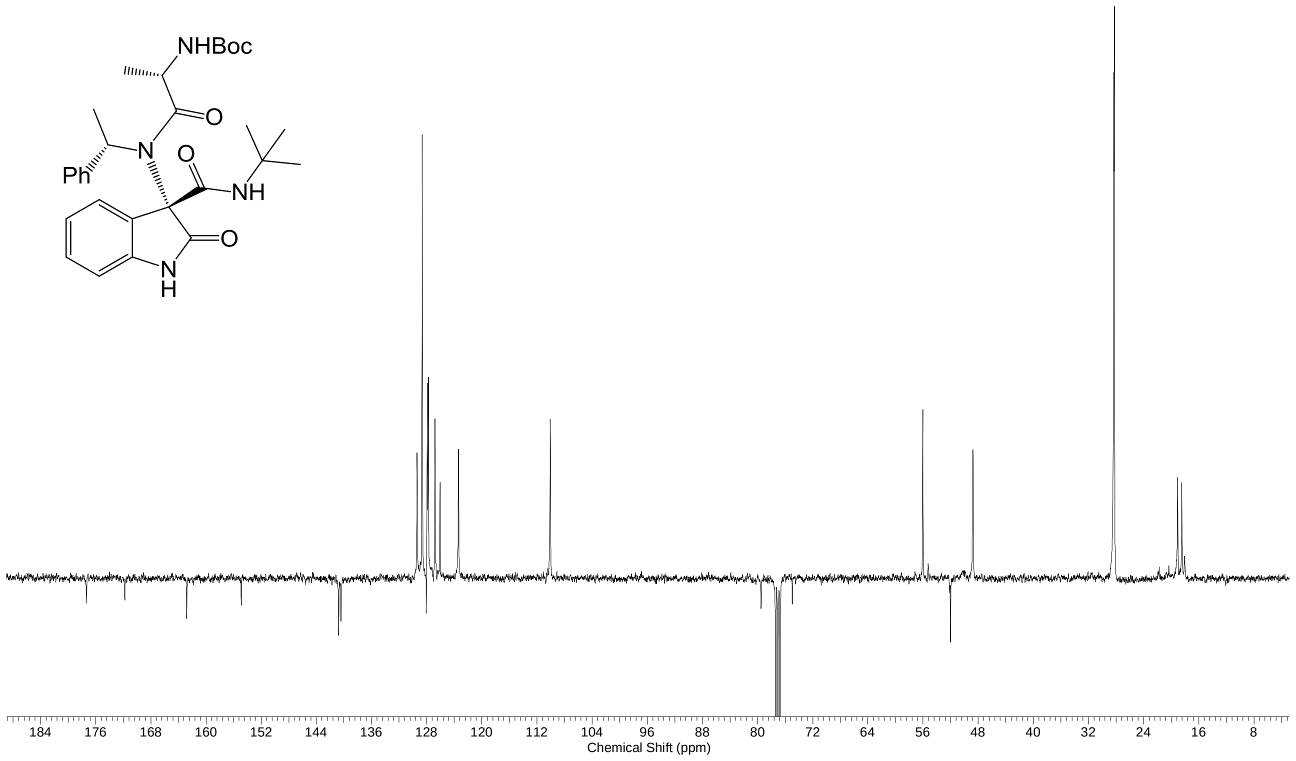

Compound **12a**: ^{1}H NMR (400 MHz, $CDCl_3$)

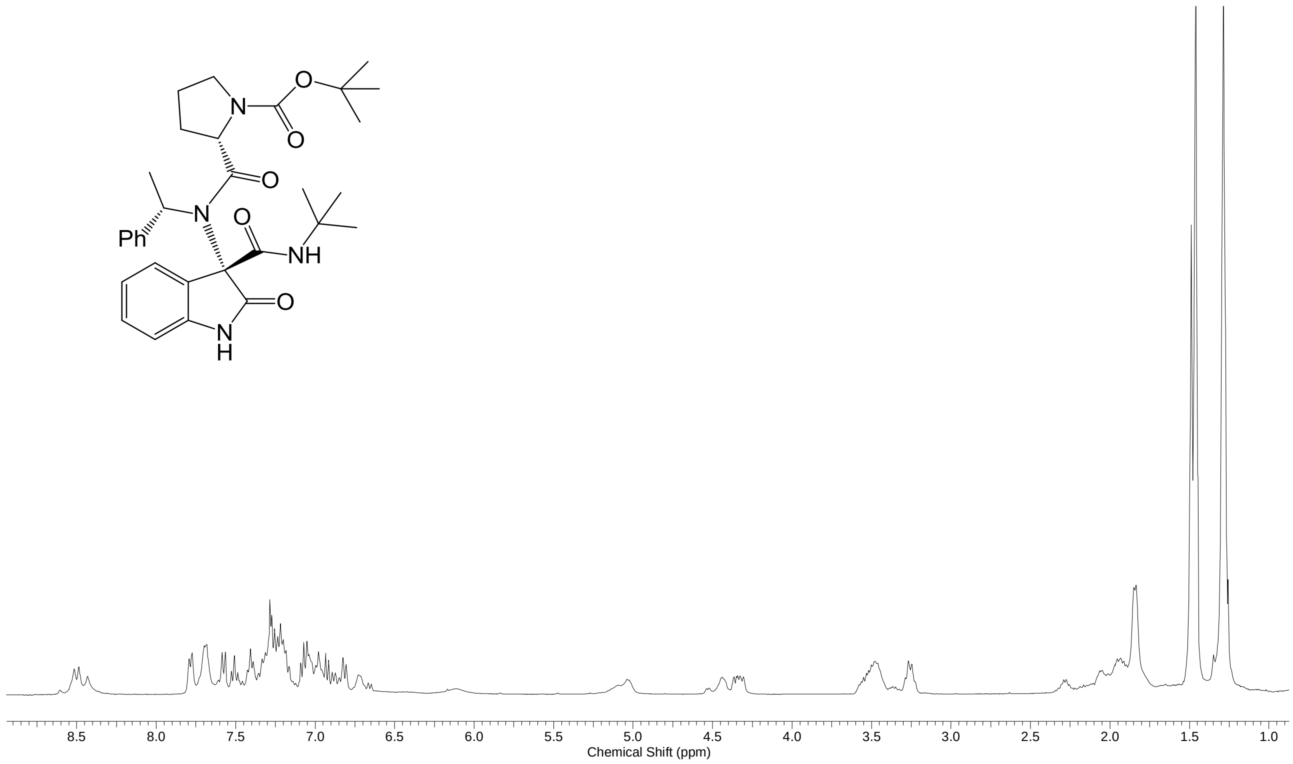

Compound **12a**: ^{13}C NMR (100 MHz, $CDCl_3$)

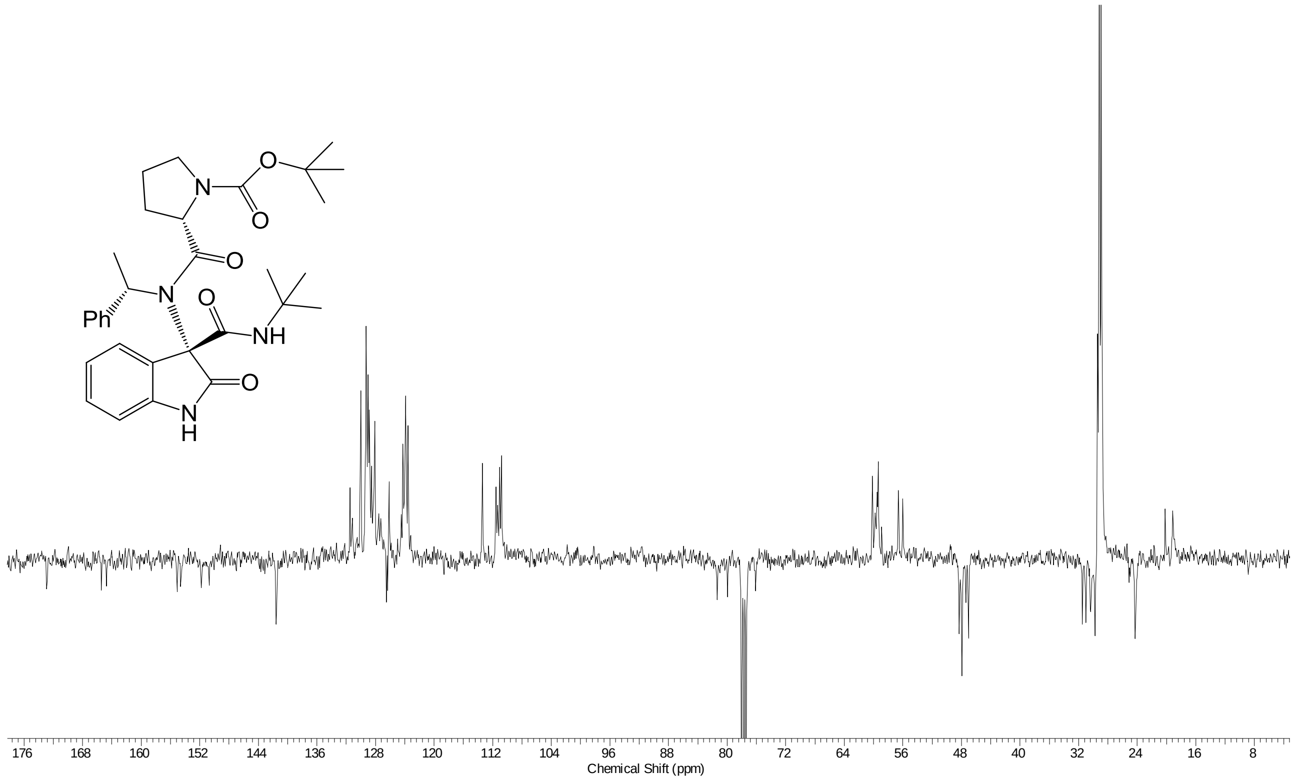

Compound **13a**: ^{1}H NMR (400 MHz, DMSO-d_6, 100 °C)

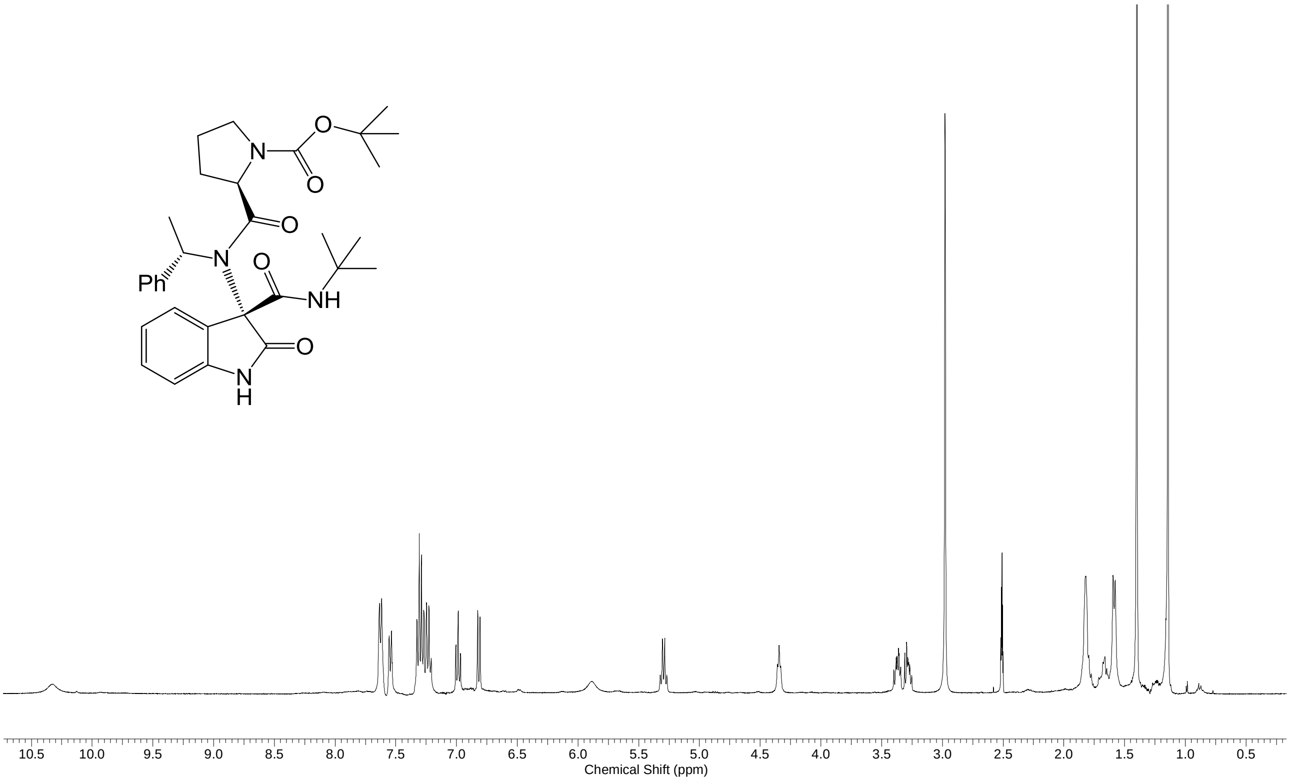

Compound **13a**: ^{13}C NMR (75 MHz, DMSO-d_6, rotameric mixture)

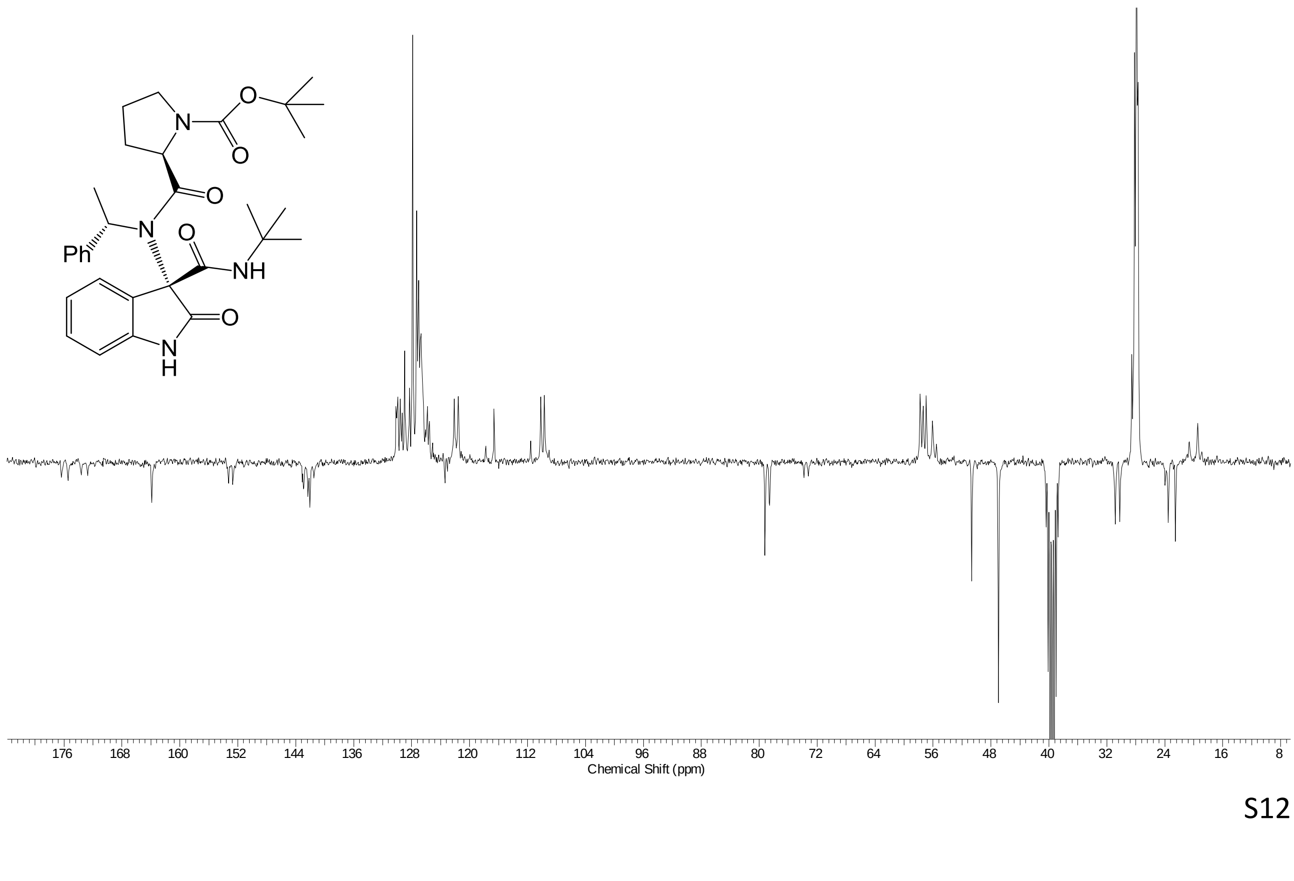

Compound **14a**: ^{1}H NMR (400 MHz, $CDCl_3$)

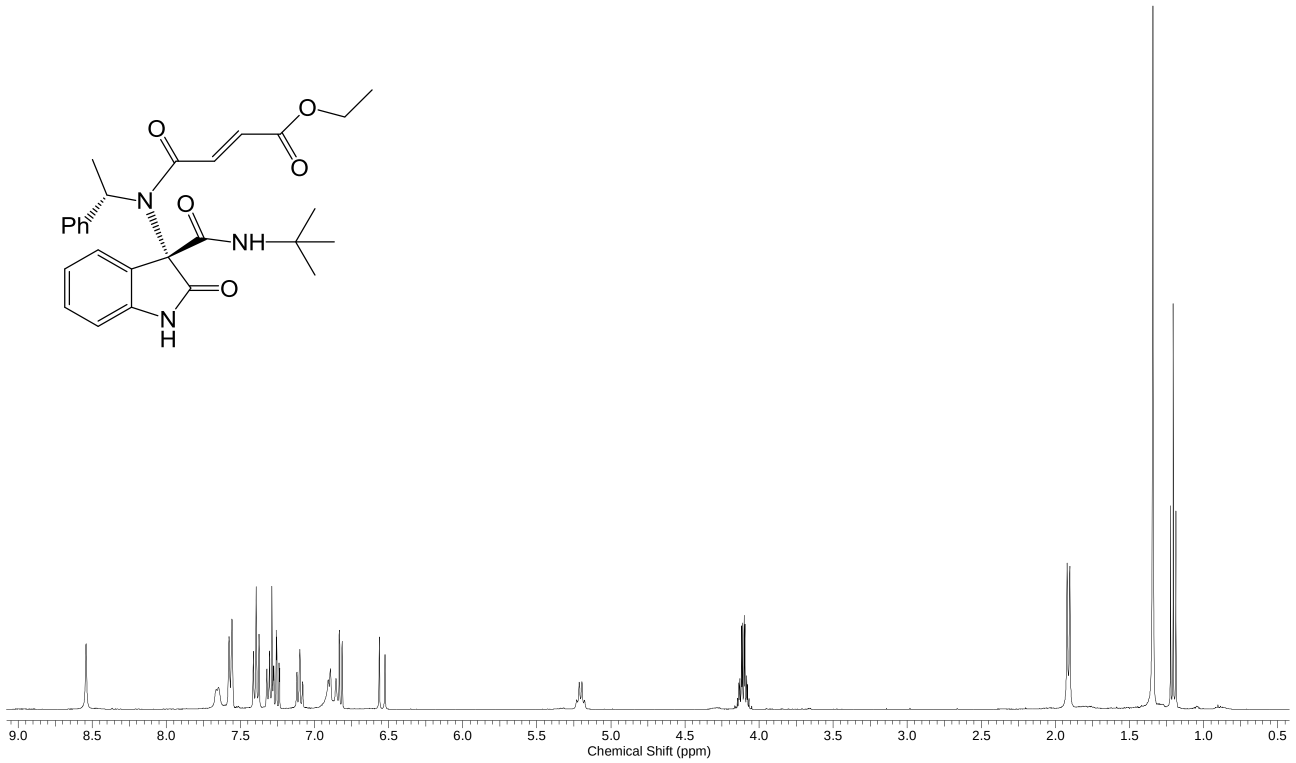

Compound **14a**: ^{13}C NMR (100 MHz, $CDCl_3$)

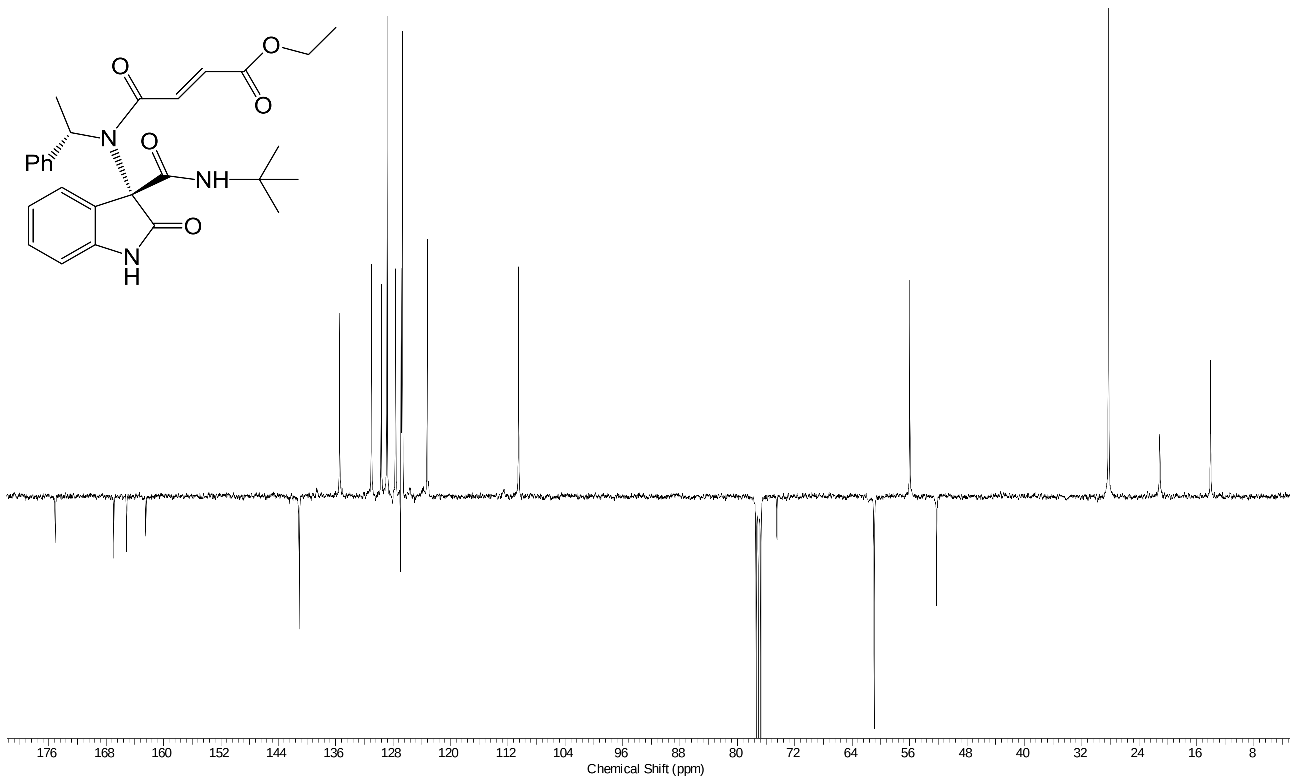

Compound **15a**: ^{1}H NMR (400 MHz, $CDCl_3$)

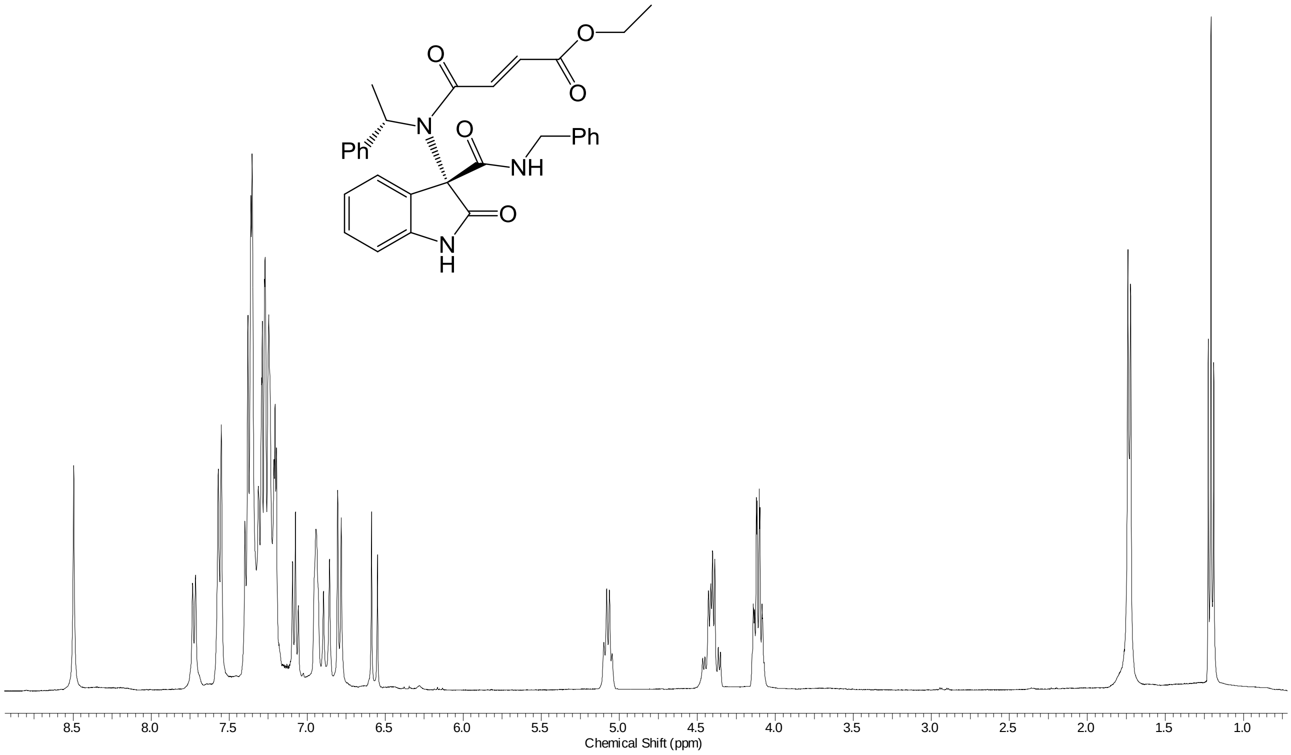

Compound **15a**: ^{13}C NMR (100 MHz, $CDCl_3$)

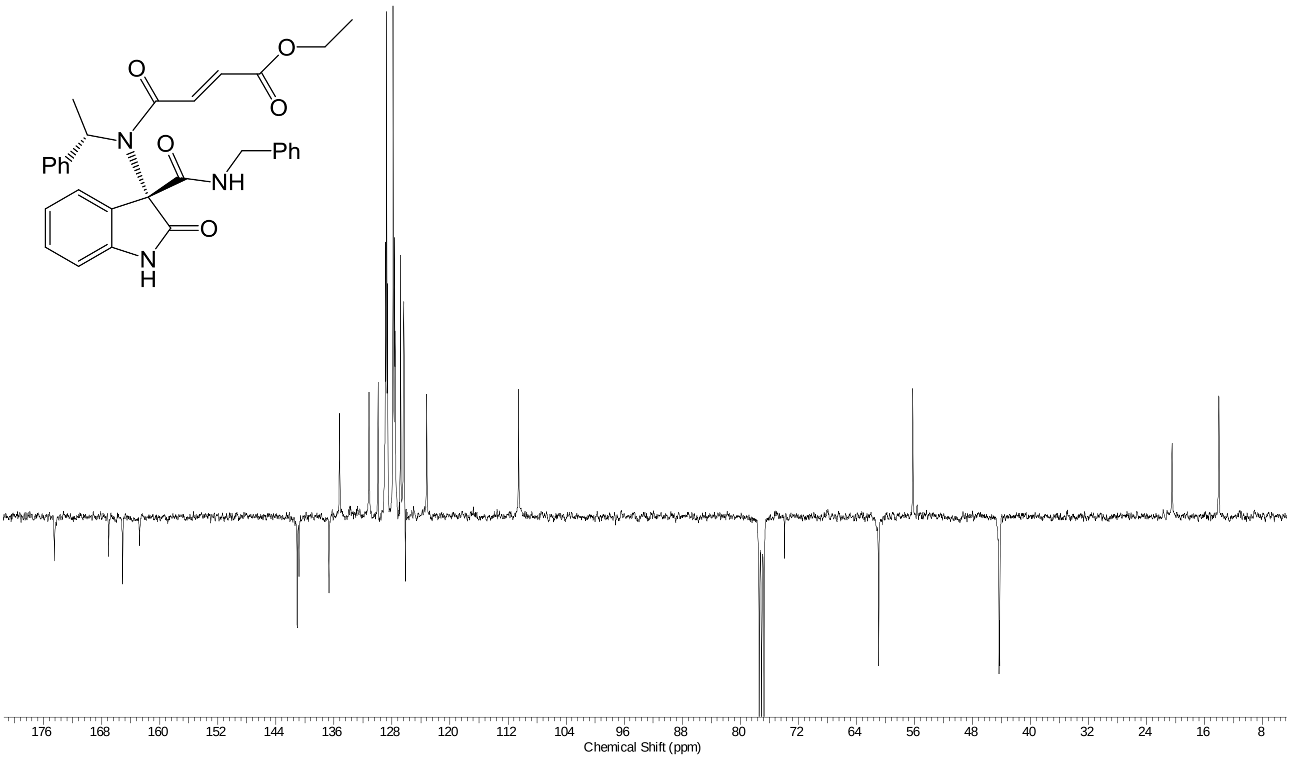

Compound **16**: ^{1}H NMR (300 MHz, $CDCl_3$, 0.15:0.85 rotameric mixture)

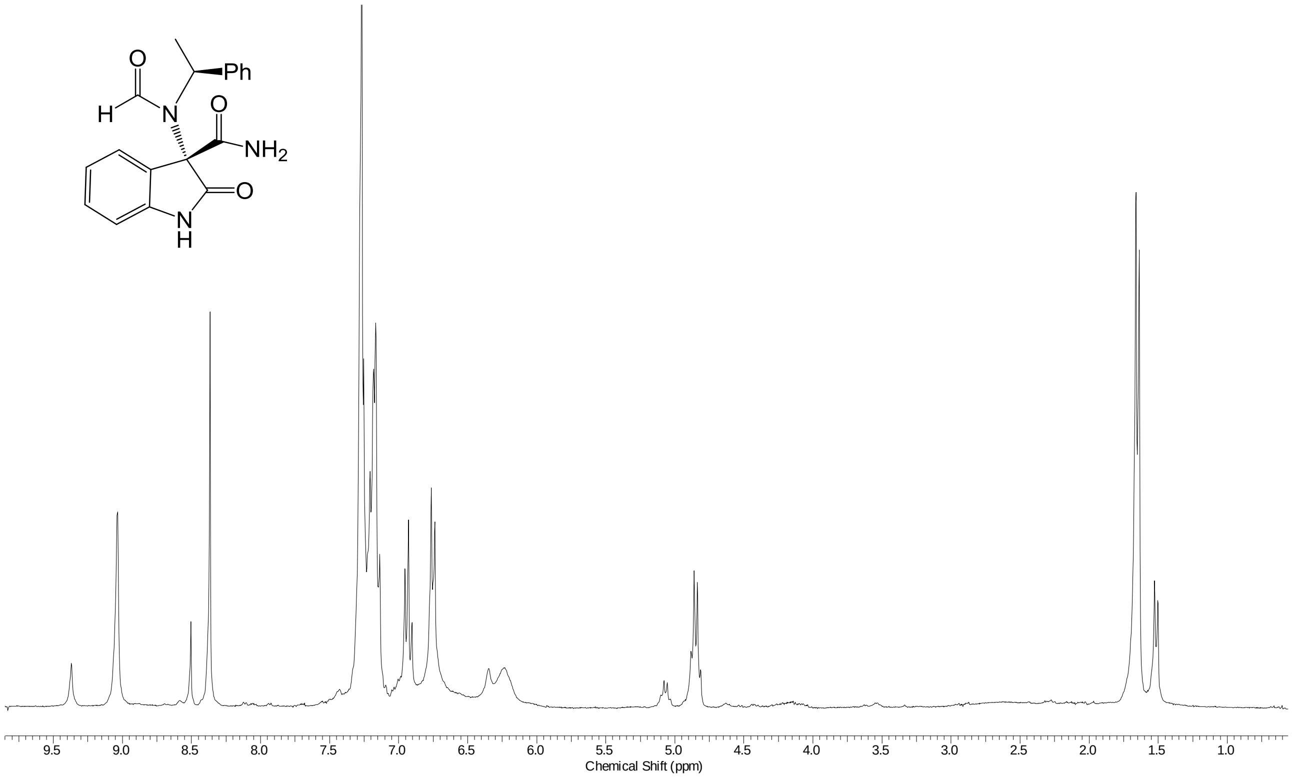

Compound **16**: ^{13}C NMR (75 MHz, $CDCl_3$)

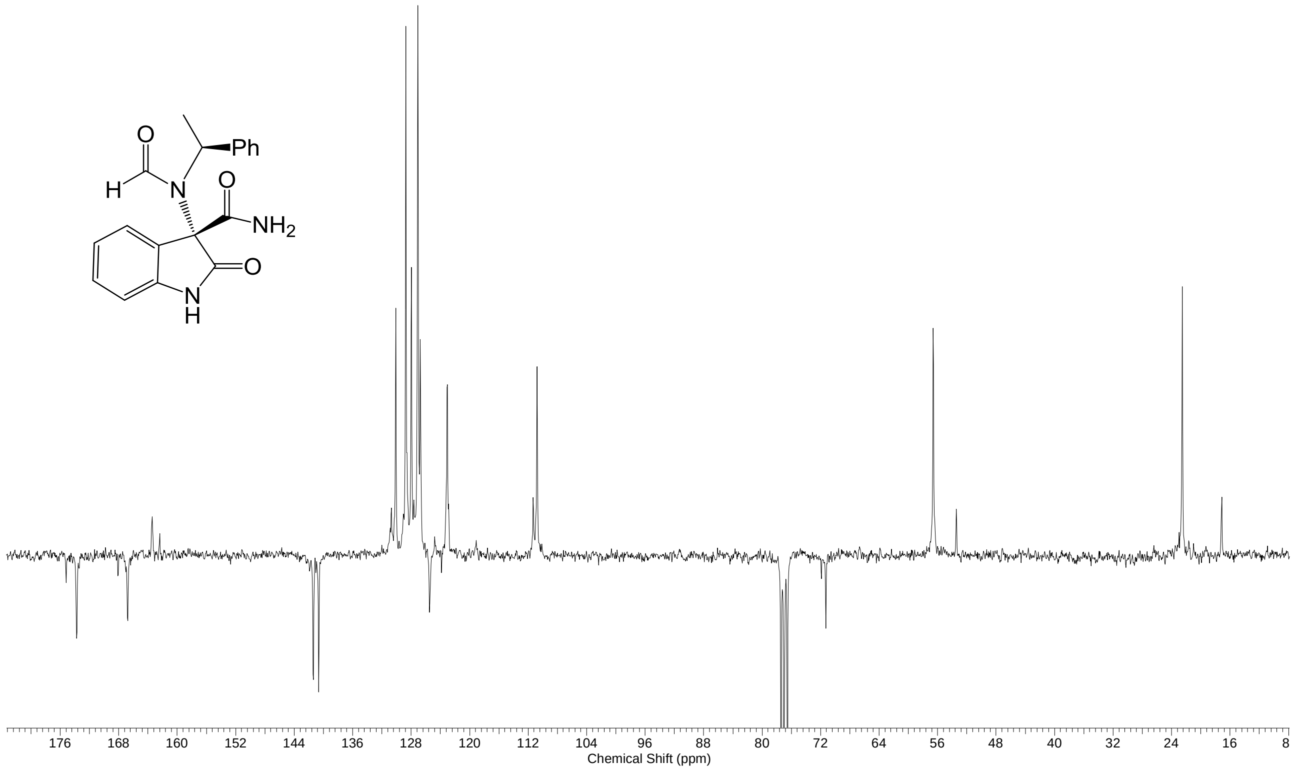

Compound **18**: ^{1}H NMR (400 MHz, $CDCl_3$)

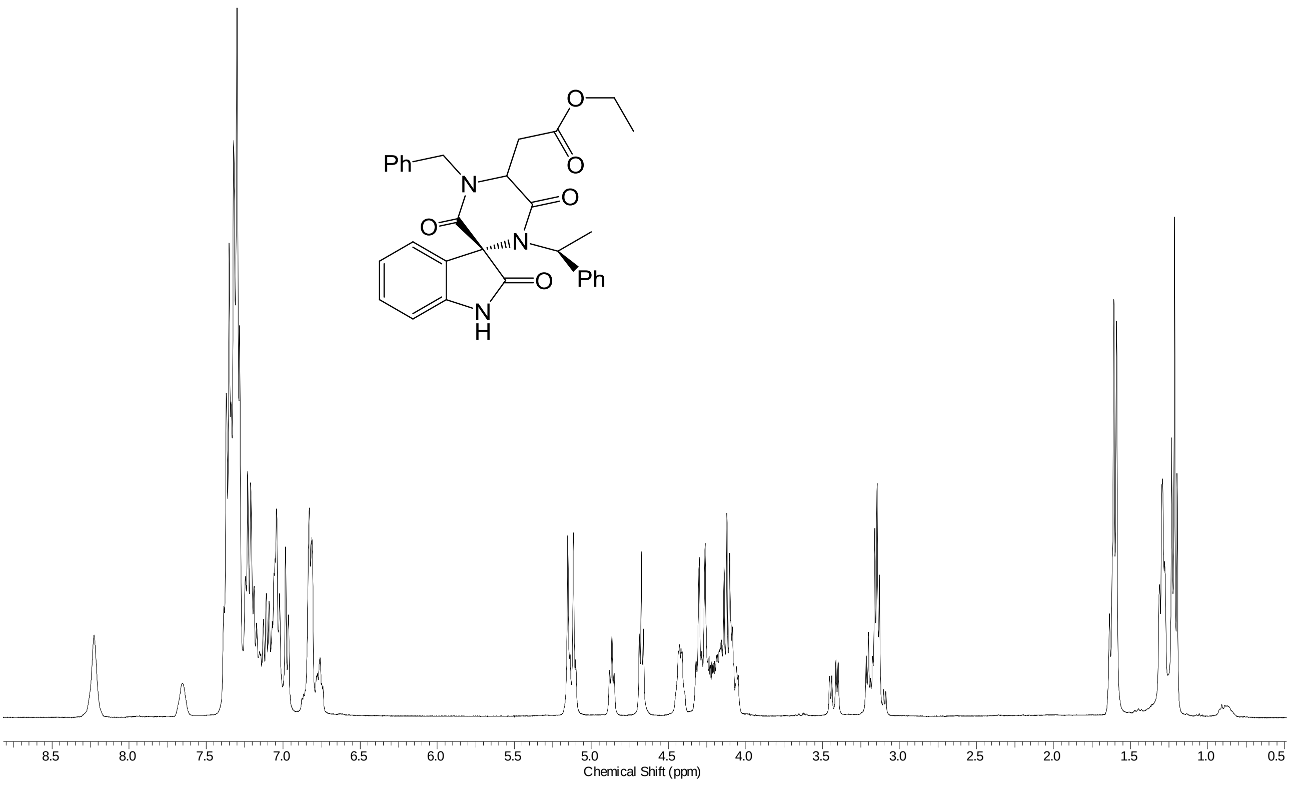

Compound **18**: ^{13}C NMR (100 MHz, $CDCl_3$)

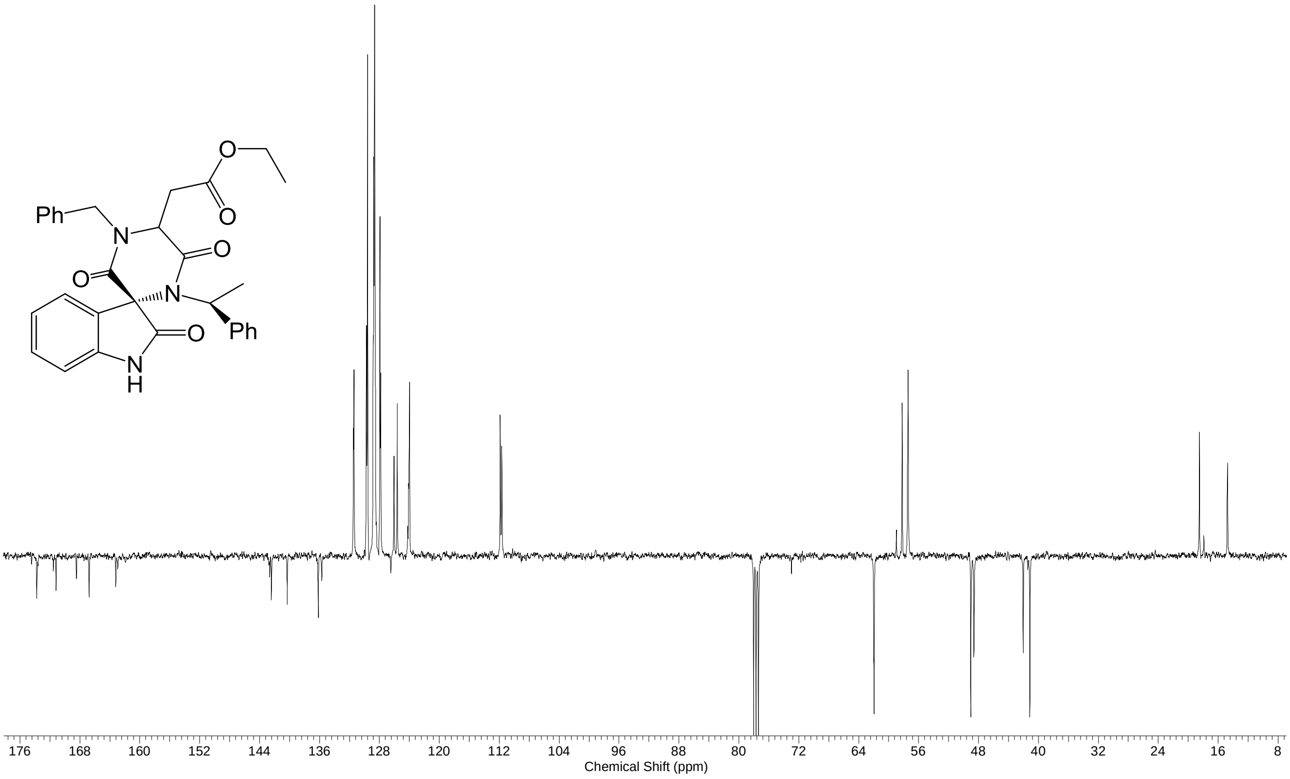

Supplement: File 2 — General methods and copies of NMR spectra for all new compounds. [file Beilstein_J_Org_Chem-10-1383-s002.pdf]
